# Genomic epidemiology of *Salmonella* Typhimurium in Guizhou, China (2019–2023): high prevalence of MDR, plasmid-associated resistance, and shared genomic lineages

**DOI:** 10.3389/fmicb.2026.1787569

**Published:** 2026-09-09

**Authors:** Ge Zhu, Lv You, Limei Wu, Yanmin Liu, Deyu Li, Junhua Wang, Shijun Li, Hui Liu, Xiaoyu Wei

**Affiliations:** 1 Laboratory of Bacterial Disease, Experimental Center, Guizhou Provincial Center for Disease Control and Prevention, Guiyang, China; 2 The Key Laboratory of Environmental Pollution Monitoring and Disease Control, Ministry of Education, School of Public Health, Guizhou Medical University, Guizhou, China; 3 Institute of Public Health and Preventive Medicine, Guizhou Provincial Center for Disease Control and Prevention, Guiyang, China

**Keywords:** genomic epidemiology, multidrug resistance, phylogenetic analysis, plasmids, *Salmonella* Typhimurium, whole-genome sequencing

## Abstract

*Salmonella* Typhimurium (*S*. Typhimurium) is a leading cause of bacterial gastroenteritis worldwide, and its escalating antimicrobial resistance (AMR) poses a critical threat to global public health. Nevertheless, epidemiological and genomic data for this pathogen in Guizhou Province remain limited. We integrated phenotypic and genomic analyses of 103 clinical *S*. Typhimurium isolates, collected from nine cities between 2019 and 2023, to characterize their genetic backgrounds, resistance mechanisms, and evolutionary dynamics. The isolates exhibited persistently high resistance to conventional antibiotics. Multidrug resistance (MDR) was universal (100% during 2019–2021), with extensively drug-resistant (XDR) isolates emerged in 2020. Substantial resistance to first-line antibiotics was also observed, including ciprofloxacin (16.5%), ceftazidime (19.4%), cefotaxime (36.9%), and azithromycin (12.6%). Whole-genome sequencing (WGS) identified 61 antimicrobial resistance genes (ARGs) and six distinct point mutations. Efflux pump-associated genes were widely distributed, and ARGs conferring tetracyclines and sulfonamides were detected at high frequencies. Resistance genotypes correlated well with phenotypes for azithromycin, ceftazidime, ampicillin, chloramphenicol, trimethoprim-sulfamethoxazole, and tetracycline (*Kappa*: 0.48-0.74). Twenty plasmid replicons were detected, with IncF (24.3%), IncI1-I (15.5%), and IncHI2 (13.6%) being the most prevalent. Notably, ARGs associated with resistance to critically important antibiotics (such as *qnr* variants, *bla_*CTX*–*M*_* and *bla_*CMY*–2_*, and *mphA/mrx*) were frequently predicted to locate on plasmid-associated contigs. Plasmid diversity expanded significantly over the five-year period, particularly in economically developed cities. Molecular typing identified ST19 and its subtypes, cgST121877 (30.1%) and cgST59516 (12.6%), as the dominant clones, suggesting pronounced regional clonal expansion. Furthermore, phylogenetic analysis revealed close genetic relatedness between Guizhou clinical isolates and human- and livestock-derived isolates from other Chinese provinces. These findings highlight the cross-regional and cross-species circulation of shared genomic lineages, suggesting complex ecological networks that may involve the food supply chain. Implementing an integrated “One Health” surveillance strategy is critical to track and limit the circulation of these resistant lineages and mitigate the associated public health threat.

## 1 Introduction

*Salmonella* Typhimurium (*S*. Typhimurium) is among the most prevalent *Salmonella* serovars responsible for human infections worldwide (Li et al., 2020; Simpson et al., 2018; Tobolowsky et al., 2022). In recent years, rising living standards and shifting dietary habits have increased the consumption of animal-derived foods, contributing to a concomitant rise in *Salmonella* infection rates. In Europe, *S*. Typhimurium is strongly associated with food contamination, particularly in chicken and pork products (Mkangara, 2023). In Africa, it is a primary driver of invasive non-typhoidal *Salmonella* (iNTS) disease (Van Puyvelde et al., 2023). In North America, although less frequent than its monophasic variant (*Salmonella* 1,4,[5],12:i:-), *S*. Typhimurium remains a significant foodborne pathogen, often causing large outbreaks linked to restaurants and food processing facilities (Delahoy et al., 2023; Zhang et al., 2019). Similarly, in China, *S*. Typhimurium consistently ranks among the top two most frequently isolated serovars from clinical diarrheal samples, indicating the highest infection incidence (Shen et al., 2020). Globally, the incidence of *Salmonella* infections remains high, particularly in developing countries and regions, where epidemiology is closely tied to geographic, climatic, and cultural factors.

Guizhou Province, located in southwestern China, has a distinctive geography, climate, and diverse dietary habits. These factors potentially create a unique ecological niche that facilitates *S*. Typhimurium transmission and the accelerated evolution of ARGs. Regional surveillance indicates that, since 2018, *S*. Typhimurium has surpassed *S*. Enteritidis as the predominant serovar among human-derived *Salmonella* isolates in this region (Wei et al., 2023). However, research on the resistance characteristics and molecular mechanisms of *S*. Typhimurium in Guizhou remains limited, posing a potential threat to local public health. Consequently, a comprehensive evaluation integrating phenotypic resistance landscapes and whole-genome characteristics of regional *S*. Typhimurium isolates is imperative. This approach will not only decipher the driving resistance mechanisms but also provide a critical scientific baseline for formulating tailored and effective infection control frameworks.

Antimicrobial therapy remains the cornerstone for managing severe *Salmonella* infections. However, the rising prevalence of AMR poses significant challenges, creating a critical bottleneck in the clinical management of salmonellosis (Aleksandrowicz et al., 2023; McDermott et al., 2018). The global spread of MDR *S*. Typhimurium is particularly alarming. The most common resistance patterns are ASSuT (ampicillin + streptomycin + sulfonamides + tetracycline) and ACSSuT (ampicillin + chloramphenicol + streptomycin + sulfonamides + tetracycline), which are widespread and increasing in many regions (San Roman et al., 2018; Wang et al., 2019). Surveillance in Guizhou Province between 2013 and 2018 showed an MDR rate of 88.1% for *S*. Typhimurium (Wei et al., 2023). More importantly, *S*. Typhimurium isolates have recently emerged with resistance to fluoroquinolones, cephalosporins, and macrolides (azithromycin), which are first-line therapies recommended by the World Health Organization (WHO). A study of *Salmonella* from retail beef and poultry processing environments revealed *S*. Typhimurium isolates resistant to ciprofloxacin, cephalosporins, and azithromycin (Osivand et al., 2025). Furthermore, analysis of 1,874 clinical *S*. Typhimurium isolates collected across China from 2020 to 2023 demonstrated persistently high resistance to nalidixic acid, streptomycin, and tetracycline. Crucially, these domestic isolates exhibited substantially higher resistance to third-generation cephalosporins (3GCs) than those reported in Europe and the United States, and resistance to azithromycin and meropenem was also documented (Sun H. et al., 2025).

In recent years, advancements in high-throughput sequencing technologies have rendered WGS combined with bioinformatic analysis indispensable for AMR research in bacteria (Galhano et al., 2021). Deep sequencing of bacterial genomes enables systematic characterization of ARG profiles, plasmid content, and genetic variations, dramatically enhancing our understanding of drug resistance mechanisms. Furthermore, WGS facilitates exploration of horizontal ARG transfer and circulation within bacterial populations, providing new insights for controlling the spread of resistant isolates.

Analysis of ARGs is essential for unraveling the molecular basis of resistance, and WGS enables their accurate identification, clarifying the link between specific genes and phenotypic resistance. For instance, genes such as *mphA* and *mcr-1* are closely related to resistance to azithromycin and colistin, respectively (Wang et al., 2023; Yang et al., 2022). The combination and variable expression of these ARGs can contribute to complex resistance phenotypes. Therefore, investigating ARGs not only informs targeted therapeutic strategies but also aids in predicting the future evolution of resistance.

Plasmids, as major mobile genetic elements (MGEs) in bacteria, are pivotal drivers in the spread of ARGs (Lin et al., 2025). They drive resistance through multiple mechanisms, including carriage of ARGs, promotion of horizontal gene transfer, and conferral of MDR by harboring several ARGs on a single plasmid. Moreover, plasmids can influence gene expression and alter bacterial physiology, for example, by affecting efflux pump function and membrane permeability (Castaneda-Barba et al., 2024; Chang et al., 2022).

Core genome multilocus sequence typing (cgMLST) and core genome single nucleotide polymorphism (cgSNP) analyses are indispensable tools for delineating phylogenetic relationships and genetic variation with unprecedented precision. Systematic comparison of core genomes enables the construction of high-resolution phylogenetic trees, thereby generating hypotheses regarding the potential transmission routes and evolutionary trajectory of *S*. Typhimurium. CgSNP analysis, in particular, identifies subtle genetic differences, providing a high-resolution genomic tool to infer the resistant isolates’ origins and monitor their circulation. These approaches have been successfully implemented in numerous molecular epidemiology studies of *Salmonella*, demonstrating broad application potential (Li et al., 2025). Global surveillance networks, such as PulseNet, routinely employ high-resolution genomic methods (including cgMLST and SNP-based analyses) to track *Salmonella* outbreaks and transmission, providing a standardized framework for genomic surveillance (Nadon et al., 2017).

Despite these epidemiological insights, studies of *S*. Typhimurium from Guizhou Province remain limited. Regional research has typically focused solely on phenotypic resistance, lacking a comprehensive investigation of ARG profiles, plasmid characteristics, and genomic variation. To address this, our study integrated phenotypic data with WGS to analyze clinical isolates collected between 2019 and 2023. By systematically characterizing resistance phenotypes, ARG compositions, plasmid features, and core-genomic variations, this study establishes a robust genomic baseline. This baseline will facilitate targeted surveillance and containment of *S*. Typhimurium, ultimately strengthening public health countermeasures against the ongoing AMR crisis in Guizhou Province.

## 2 Materials and methods

### 2.1 *S. Typhimurium* isolates collection

All *Salmonella* isolates were obtained from the Chinese Pathogen Identification Network (CPIN) sentinel sites in Guizhou Province. Between January 2019 and December 2023, diarrheal stool samples were collected from patients at 109 sentinel hospitals across nine cities (prefectures). The inclusion criteria were: (i) three or more loose stools within 24 hours, with changes in stool consistency (such as watery, mucoid); or (ii) fever (≥ 38°C) accompanied by symptoms such as headache, chills, or malaise. All samples were from sporadic community-acquired infections. Patients with hospital-acquired infections or a history of hospitalization within the previous 30 days were excluded. Only one isolate per patient was included to avoid duplication.

All *Salmonella* isolates recovered from eligible patients during the study period were consecutively submitted to the Guizhou Provincial Center for Disease Control and Prevention laboratory for confirmatory identification using the MALDI-TOF MS system MS1000 (Autobio Diagnostics Co., Ltd., China). All confirmed isolates were serotyped by slide agglutination for O and H antigens (SSI, Denmark) according to the White-Kauffmann-Le Minor scheme (Guibourdenche et al., 2010).

### 2.2 Antimicrobial susceptibility testing (AST)

Antimicrobial susceptibility was evaluated using the broth microdilution method for 17 antimicrobials from 11 classes (CLSI2023 M100) (Supplementary Data S1): macrolides [azithromycin (AZM)]; quinolones and fluoroquinolones [ciprofloxacin (CIP), nalidixic acid (NA)]; cephems [ceftazidime (CAZ), cefotaxime (CTX)]; carbapenems [meropenem (MEM), ertapenem (ETP)]; lipopeptide [colistin (CL)]; tetracyclines [tetracycline (TE), tigecycline (TGC)]; penicillin [ampicillin (AM)]; phenicol [chloramphenicol (C)]; sulfonamides [trimethoprim-sulfamethoxazole (SXT)]; aminoglycosides [streptomycin (STS), amikacin (AN)]; and β-lactamase inhibitor [ampicillin/sulbactam (SAM), ceftazidime/avibactam (CZA)]. The minimal inhibitory concentration (MIC) was determined using a customized AST plate CHNENF (Thermo Fisher Scientific, United States). *Escherichia coli* ATCC 25922 was used as the quality control strain. MDR was defined as resistance to at least three different antibiotic classes, and XDR was defined as susceptibility to only one or two classes (Magiorakos et al., 2012).

### 2.3 Genomic DNA extraction and WGS

Genomic DNA was extracted from the isolates using a commercial kit (Baiju Technologies, China) and quantified using a Qubit 4 Fluorometer with the Qubit dsDNA HS Assay Kit (Thermo Scientific, United States). All DNA samples were aliquoted and stored at −20 °C for downstream analyses.

DNA libraries were constructed using the MGIEasy FS DNA Library Prep Set (MGI, China) and were paired-end (PE100) sequenced on the MGISEQ-2000RS platforms (MGI). Raw FASTQ files were generated and subjected to quality control using FastQC v0.11.7.^1^ Low-quality reads and those containing excessive ambiguous bases (N) were removed. The remaining high-quality reads were assembled *de novo* using the MGAP (Microbial Genome Assembly Pipeline) module on the MGI bioinformatics platform (MGAP assembly v2.1.0). Assembly quality was assessed with Quast v4.6.3 (e.g., N50, GC content, assembly size, sequencing depth, and contig counts; Supplementary Data S2).

### 2.4 Prediction of ARGs and plasmids

ARGs and point mutations were screened using the Comprehensive Antibiotic Resistance Database (CARD),^2^ with the “strict” paradigm, applying thresholds of > 80% for both identity and coverage. Plasmid replicons were identified using PlasmidFinder v2.1 (identity > 95%, coverage > 60%). To delineate plasmid-associated resistance, we predicted plasmid contigs using PlasmidHunter^3^ ((Tian et al., 2024) with default parameters and subsequently screened for ARGs on these contigs (> 80% identity and coverage).

### 2.5 CgMLST analysis

#### 2.5.1 The prevalence of cgMLST from Guizhou

Core-genome sequence types (cgSTs) were determined with cgMLSTFinder v1.2.0^4^ and integrated with epidemiological information for comprehensive analysis.

#### 2.5.2 Neighbor-joining tree (NJ tree) analysis based on cgMLST profiles

To investigate genetic relationships, a neighbor-joining (NJ) tree was constructed using IQ-TREE v2.4.0^5^ based on the cgMLST allele profile. We integrated epidemiological information, sequence types (STs; determined via PubMLST), resistance phenotypes, ARGs, and plasmid replicons to analyze the population structure, characterize the distribution of ARGs and plasmids, and examine genetic relationships across different clusters.

#### 2.5.3 Minimum spanning tree (MST) analysis based on 3002 allele profiles

Epidemiological metadata were retrieved from EnteroBase and systematically filtered to exclude isolates with incomplete records (e.g., missing isolation year, country, or source) or those without publicly accessible genome sequences in NCBI. We included 168 *S*. Typhimurium isolates in this study, and their whole-genome sequences were downloaded from NCBI using respective accession numbers (Supplementary Data S3).

The 168 isolates, collected between 2012 and 2023, originated from seven countries (including China, the USA, and the UK) and diverse sources: human; animal (e.g., aquatic, companion, wild); food (e.g., pork, beef, chicken, duck); and environment (e.g., water, farm).

The 271 genomes (including 103 from Guizhou and 168 on NCBI) were analyzed with cgMLSTFinder v1.2.0 to obtain 3002 allele profiles (Supplementary Data S4). A minimum spanning tree (MST) was then constructed using GrapeTree v1.5.0^6^ with the MSTreeV2 algorithm to analyze genetic relationships, population structures, and potential transmission pathways.

### 2.6 CgSNP analysis

To further investigate evolutionary relationships, a cgSNP analysis was conducted for isolates within cluster A1 in the MST. This analysis included isolates from Guizhou and other Chinese provinces (Beijing, Shanghai, Guangdong, Zhejiang, Sichuan, Chongqing, Yunnan, Jilin, and Guangxi) with diverse sources (livestock, animals, environment, and human) (Supplementary Data S5).

The cgSNP analysis was performed using the BAIYI MicroGeno Platform (v5.4; Hangzhou Baiyi Technology Co., Ltd.).^7^ The *S*. Typhimurium LT2 genome (GenBank: NC_003197) was used as the reference. SNPs were called using Snippy v4.6.0^8^ with default settings, retaining only positions with base quality ≥ Q30 and coverage ≥ 10 × ; SNPs in repetitive or low-complexity regions were excluded. The resulting core-genome alignment was approximately 4.2 Mb, containing approximately 15,000 high-quality SNPs. Potential recombinant regions were identified and removed using Gubbins v3.3.5^9^ to ensure accurate phylogenetic inference. Maximum-likelihood (ML) phylogenetic trees were constructed with IQ-TREE v2.4.0. The best-fit substitution model (F81 + F + ASC) was selected using ModelFinder Plus.

### 2.7 Statistical analysis

All statistical analyses were performed using R version 4.3.2 (R Foundation for Statistical Computing, Vienna, Austria). Temporal trends in resistance rates for each antibiotic across the five years (2019–2023) were assessed using the Cochran-Armitage trend test. Temporal comparisons of resistance rates between 2021 and 2022 were conducted using the chi-square test or Fisher’s exact test (for expected counts < 5). Agreement between resistance phenotypes and genotypes was assessed by calculating sensitivity, specificity, and Cohen’s *Kappa* with 95% confidence interval (*CI*). Sensitivity and specificity were computed from 2 × 2 contingency tables, with 95% *CI* estimated using the Clopper-Pearson exact method. *Kappa* values and their 95% confidence intervals were calculated using the psych package. A *Kappa* value of ≥ 0.75 indicated excellent agreement; 0.40–0.75, good agreement; and ≤ 0.40, poor agreement. A *P*-value of < 0.05 was considered statistically significant.

## 3 Results

### 3.1 Spatiotemporal distribution of *S.* Typhimurium

**FIGURE 1:**
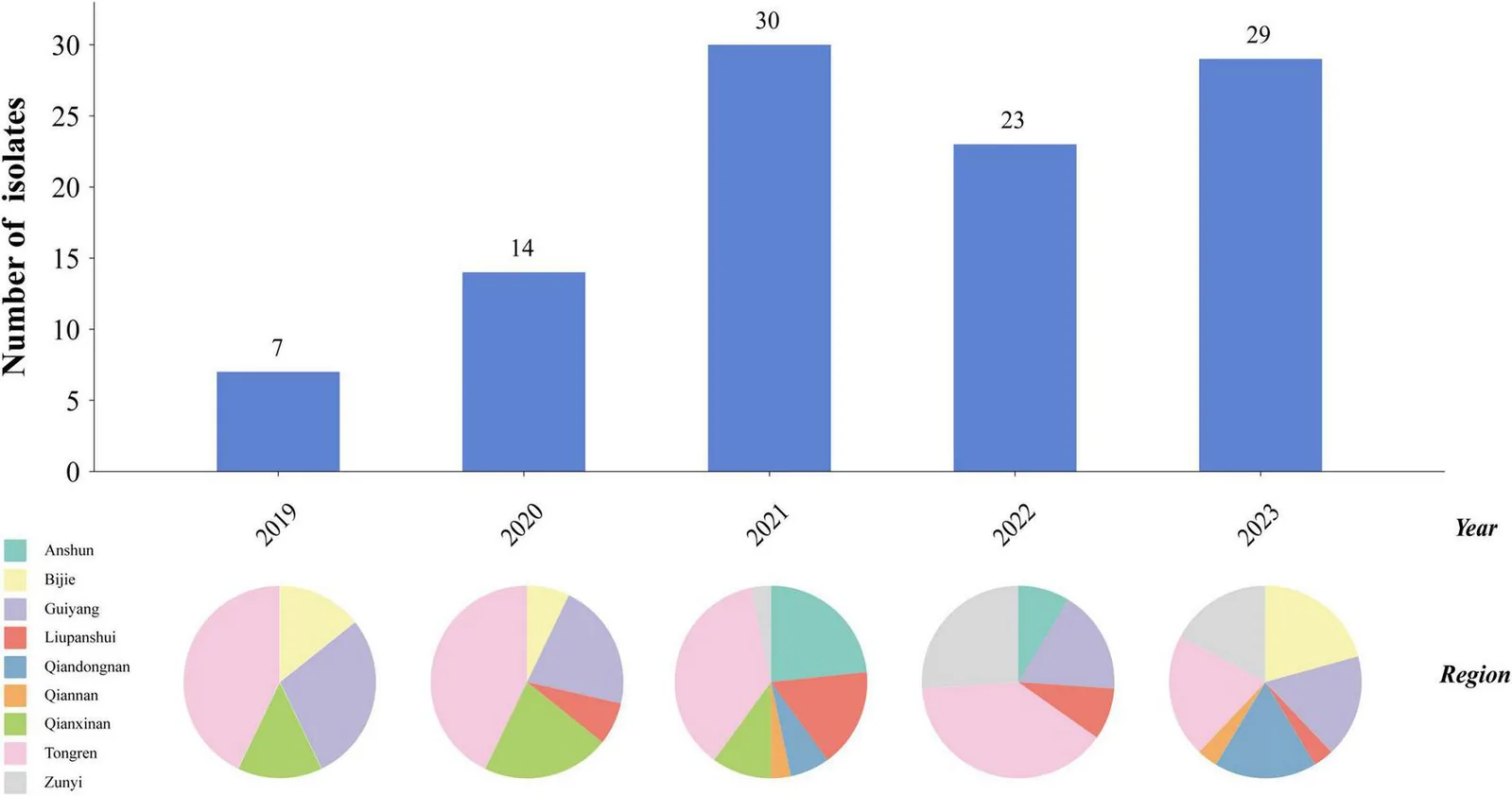
Distribution of 103 *S*. Typhimurium isolates from Guizhou Province. The upper panel shows the number of isolates in Guizhou from 2019 to 2023. The lower panel displays the distribution of isolates collected from the nine cities (prefectures) in Guizhou each year.

Between 2019 and 2023, a total of 931 *Salmonella* isolates were recovered from diarrheal patients in Guizhou Province, of which 103 (11.1%) were identified as *S*. Typhimurium. Temporally, the annual proportions of *S*. Typhimurium among all *Salmonella* isolates remained relatively stable, ranging from 10.0 to 13.2% across the 5 years. Spatially, the highest proportions of *S*. Typhimurium were observed in Bijie (8/33, 24.2%), Qianxinan (7/32, 21.9%), and Anshun (9/45, 20.0%) (Supplementary Data S6). Among the 103 *S*. Typhimurium isolates, 7 (6.8%) were collected in 2019, 14 (13.6%) in 2020, 30 (29.1%) in 2021, 23 (22.3%) in 2022, and 29 (28.2%) in 2023 (Figure 1). The largest numbers were recovered from Tongren (35/103, 34.0%), Guiyang (14/103, 13.6%), and Zunyi (12/103, 11.7%).

### 3.2 Antimicrobial resistance

**FIGURE 2:**
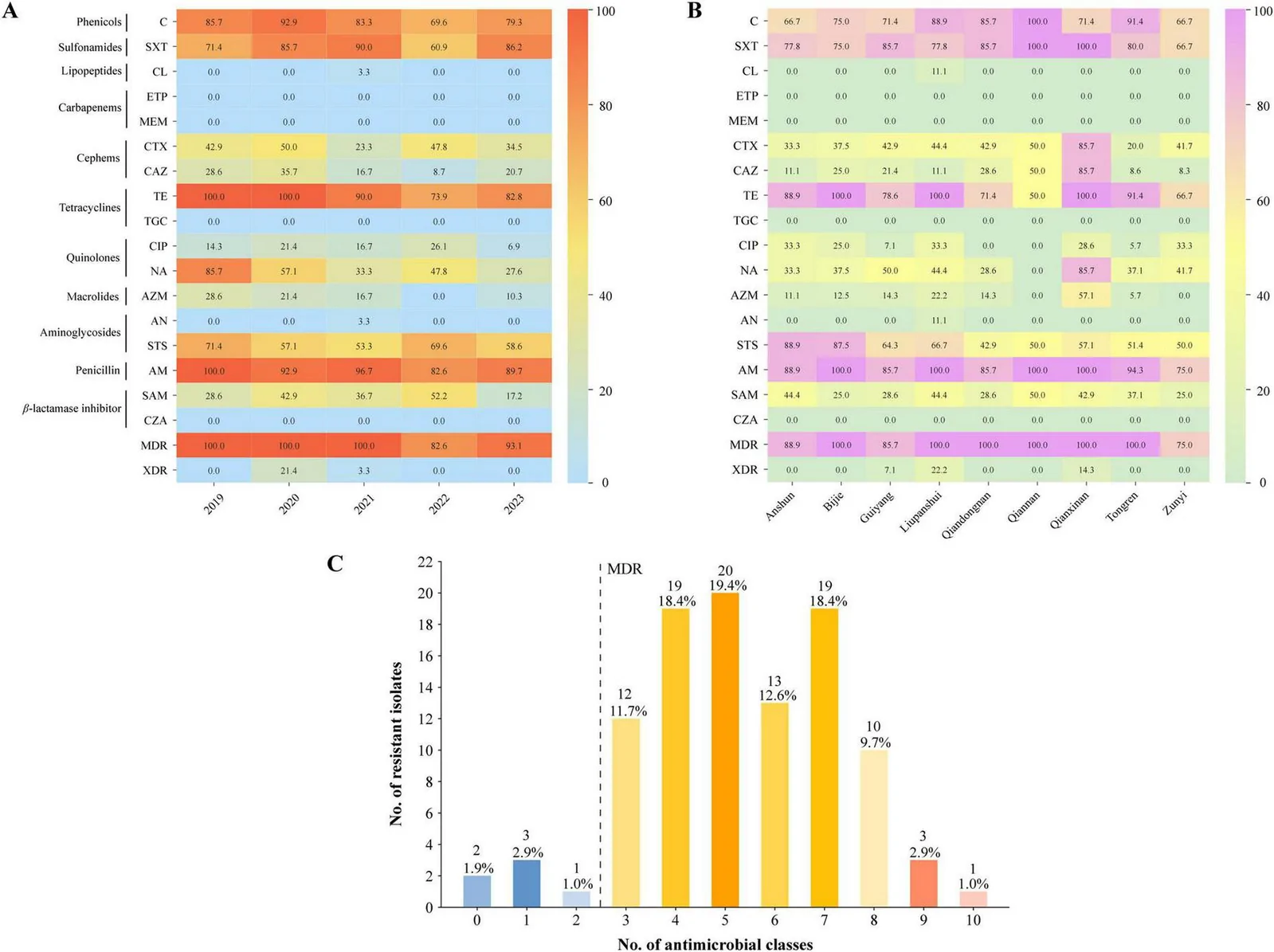
Phenotypic resistance of 103 *S*. Typhimurium isolates. **(A)** The heatmap depicts the temporal trends (2019-2023) for both the resistance rates to 17 antibiotics and the prevalence rates of MDR and XDR among *S*. Typhimurium isolates in Guizhou Province. Antibiotic abbreviations: C, chloramphenicol; SXT, trimethoprim-sulfamethoxazole; CL, colistin; ETP, ertapenem; MEM, meropenem; CTX, cefotaxime; CAZ, ceftazidime; TE, tetracycline; TGC, tigecycline; CIP, ciprofloxacin; NA, nalidixic acid; AZM, azithromycin; AN, amikacin; STS, streptomycin; AM, ampicillin; SAM, ampicillin/sulbactam; CZA, ceftazidime/avibactam. **(B)** The heatmap depicts the spatial distribution of resistance rates to 17 antibiotics and the prevalence rates of MDR and XDR across nine cities (prefectures) in Guizhou Province. **(C)** Distribution of antimicrobial resistance categories among *S*. Typhimurium isolates in Guizhou Province. The area to the right of the dashed line indicates MDR isolates.

The 103 *S*. Typhimurium isolates exhibited varying resistance levels to the 17 antibiotics tested, with an overall resistance rate of 98.1% (101/103). Ampicillin (94/103, 91.3%) and tetracycline (89/103, 86.4%) exhibited the highest resistance rates, followed by chloramphenicol and trimethoprim-sulfamethoxazole (both 83/103, 80.6%). The resistance rates for ciprofloxacin, ceftazidime, cefotaxime, and azithromycin were 16.5% (17/103), 19.4% (20/103), 36.9% (38/103), and 12.6% (13/103), respectively (Supplementary Data S7). One colistin-resistant isolate (SM2021076) was identified in Liupanshui (MIC = 4 μg/mL). The Cochran-Armitage trend tests revealed no significant monotonic trend in resistance rates from 2019 to 2023 across any of the 17 antibiotics tested (all *P* > 0.05). However, a transient decline in resistance rates for multiple antibiotics was observed in 2022 (Figure 2A). For the four conventional antibiotics (chloramphenicol, trimethoprim-sulfamethoxazole, tetracycline, and ampicillin), resistance rates declined in 2022 compared to 2021, with a statistically significant decrease for trimethoprim-sulfamethoxazole (χ^2^ = 22.63, *P* < 0.001). Resistance to the first-line antibiotics ceftazidime and azithromycin also declined in 2022, with azithromycin resistance dropping to zero. Additionally, resistance rates varied across the nine cities (prefectures) (Figure 2B). Overall, resistance to conventional antibiotics remained high in most regions, although Zunyi showed relatively lower rates. Among the first-line antibiotics, Qianxinan exhibited notably high resistance to both cefotaxime (6/7, 85.7%) and ceftazidime (6/7, 85.7%). Azithromycin resistance was concentrated in Qianxinan (4/7, 57.1%), with low or no resistance in other regions. Ciprofloxacin resistance was absent in Qiandongnan and Qiannan but was higher in Anshun, Liupanshui, and Zunyi (all 33.3%). The MDR rate exceeded 85% in all regions except Zunyi (9/12, 75.0%), while XDR isolates were only in Liupanshui (2/9, 22.2%), Qianxinan (1/7, 14.3%), and Guiyang (1/14, 7.1%).

The MDR rate was 94.2% (97/103). Resistance to five antibiotic classes was the most common (20/103, 19.4%), followed by resistance to seven and four classes (both 19/103, 18.4%) (Figure 2C). The dominant AMR pattern across the province was C + SXT + TE + STS + AM (13/103, 12.6%), followed by C + SXT + TE + AM (11/103, 10.7%) (Supplementary Data S7). These two patterns were also the most common in seven of the cities (prefectures), except Qianxinan and Qiannan (Table 1). Notably, the two dominant AMR patterns in Qianxinan both included 3GCs (ceftazidime and cefotaxime), whereas those in other regions largely consisted of conventional antibiotics. The MDR rate remained at 100% from 2019 to 2021, decreased to 82.6% in 2022, and rose again to 93.1% in 2023. XDR isolates accounted for 3.9% (4/103) and were detected in 2020 (3 isolates) and 2021 (1 isolate). Additionally, one isolate from Liupanshui in 2021 exhibited co-resistance to 13 antibiotics across 10 classes (C + SXT + CL + CTX + CAZ + TE + CIP + NA + AZM + AN + STS + AM + SAM).

**TABLE 1:** Distribution of dominant AMR patterns of *S*. Typhimurium from nine cities (prefectures) in Guizhou*.

Region	AMR pattern	Number of isolates	Percent (%)
Anshun	C + SXT + TE + STS + AM	2	22.2
	C + SXT + CTX + TE + CIP + STS + AM + SAM	2	22.2
Bijie	C + SXT + TE + STS + AM	3	37.5
	C + SXT + TE + STS + AM + SAM	1	12.5
Guiyang	C + SXT + TE + AM	2	14.3
	C + SXT + CTX + TE + STS + AM + SAM	2	14.3
Liupanshui	C + SXT + TE + AM	2	22.2
	C + SXT + TE + STS + AM	2	22.2
Qiandongnan	C + SXT + TE + AM	1	14.3
	C + SXT + TE + STS + AM	1	14.3
Qiannan	C + SXT + STS + AM	1	50.0
	C + SXT + CTX + CAZ + TE + AM + SAM	1	50.0
Qianxinan	C + SXT + CTX + CAZ + TE + NA + AZM + STS + AM	1	14.3
	C + SXT + CTX + CAZ + TE + AM	1	14.3
Tongren	C + SXT + TE + AM	4	11.4
	C + SXT + TE + AM + SAM	4	11.4
Zunyi	C + SXT + TE + AM	2	16.7
	C + SXT + CTX + TE + CIP + NA + STS + AM	2	16.7

* This table lists the two dominant AMR patterns for each city (prefecture). Abbreviations: C, chloramphenicol; SXT, trimethoprim-sulfamethoxazole; CL, colistin; ETP, ertapenem; MEM, meropenem; CTX, cefotaxime; CAZ, ceftazidime; TE, tetracycline; TGC, tigecycline; CIP, ciprofloxacin; NA, nalidixic acid; AZM, azithromycin; AN, amikacin; STS, streptomycin; AM, ampicillin; SAM, ampicillin/sulbactam; CZA, ceftazidime/avibactam.

### 3.3 Prevalence of ARGs and plasmids

**FIGURE 3:**
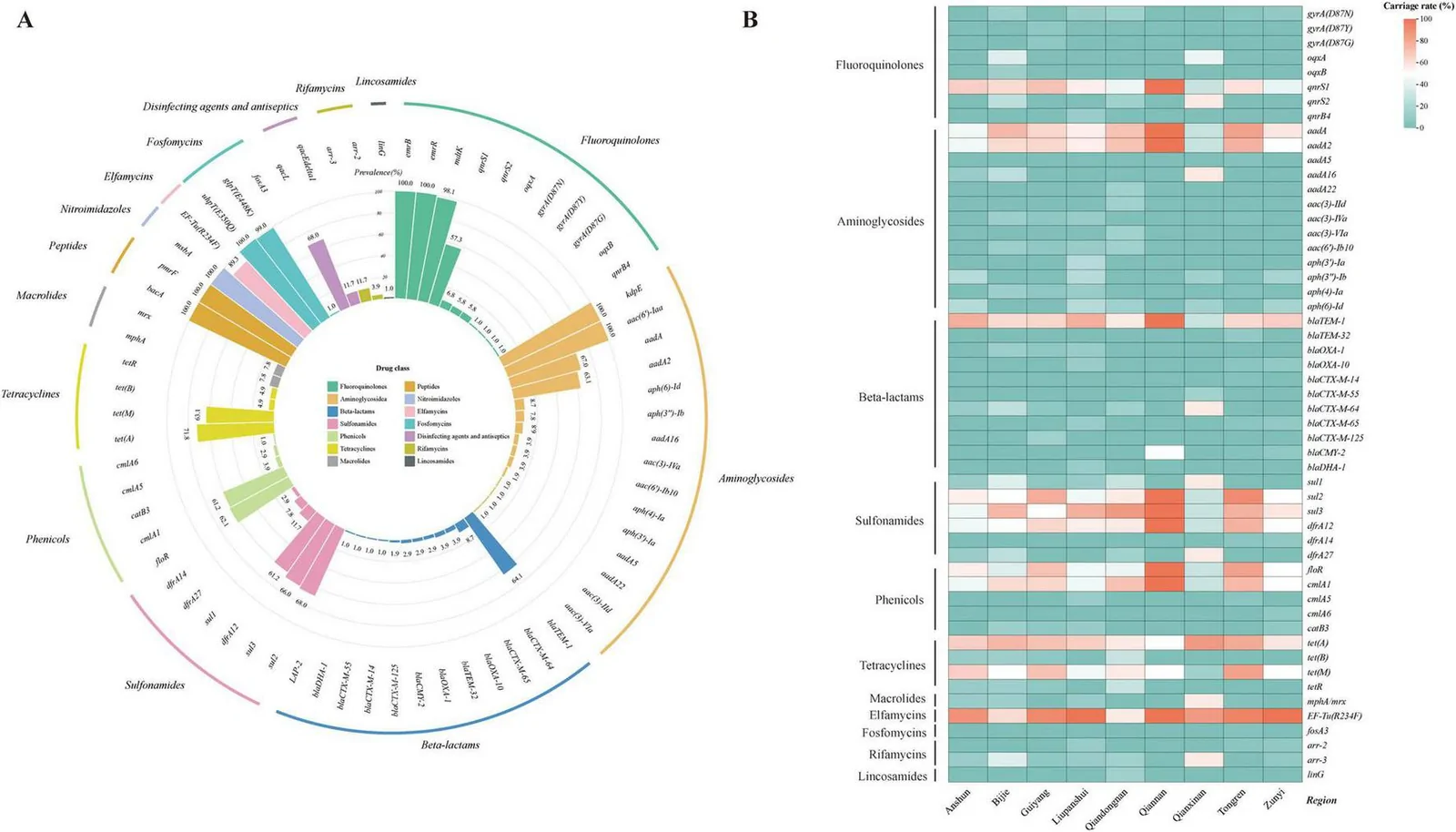
Prevalence of ARGs in *S*. Typhimurium from Guizhou Province. **(A)** Prevalence of ARGs and point mutations in 103 *S*. Typhimurium isolates. Different antimicrobial classes are color-coded, and the numbers on the outer ring of the circular histogram represent the prevalence of the corresponding ARGs. **(B)** Prevalence of ARGs across nine cities (prefectures) in Guizhou Province. The heatmap shows the carriage rate (%) of ARGs in *S*. Typhimurium isolates from each city (prefecture).

A total of 61 ARG types were detected in the 103 isolates (Figure 3A). All isolates carried efflux pump genes associated with fluoroquinolone (*emrB*, *emrR*), aminoglycoside [*kdpE*, *aac(6’)-Iaa*], and peptide (*bacA*, *pmrF*) resistance, along with the nitroimidazole resistance gene *msbA*. Several ARGs were highly prevalent within their respective classes: *qnrS1* (fluoroquinolone; 59/103, 57.3%), *aadA* (aminoglycoside; 69/103, 67.0%), *sul2* (sulfonamide; 70/103, 68.0%), *floR* (chloramphenicol; 64/103, 62.1%), *tet(A)* (tetracycline; 74/103, 71.8%), and *qacL* (disinfecting agents and antiseptics; 70/103, 68.0%). Six distinct point mutations were also detected. Among them, three were highly prevalent: the fosfomycin mutations *uhpT* (E350Q) (103/103, 100.0%) and *glpT* (E448K) (102/103, 99.0%), and the elfamycin mutation *EF-Tu* (R234F) (92/103, 89.3%). Additionally, three mutations in the *gyrA* were identified at position D87 within the quinolone resistance-determining region (*QRDR*): *gyrA* (D87N) (6/103, 5.8%), *gyrA* (D87Y) (1/103, 1.0%), and *gyrA* (D87G) (1/103, 1.0%).

Notably, this study detected multiple ARGs conferring resistance to first-line antibiotics. Among β-lactams, five ESBL *bla_*CTX*–*M*_* variants were identified with an overall carriage rate of 16.5% (17/103). *bla_*CTX*–*M*–64_* (9/103, 8.7%) and *bla_*CTX*–*M*–65_* (4/103, 3.9%) were most prevalence, followed by *bla_*CTX*–*M*–125_* (2/103, 1.9%). Each of *bla_*CTX*–*M*–14_* and *bla_*CTX*–*M*–55_* was detected in only one isolate. Additionally, the AmpC β-lactamases gene *bla_*CMY*–2_* was detected in 2.9% (3/103) of isolates. For macrolides, the azithromycin resistance genes *mphA* and *mrx* were detected at a carriage rate of 7.8% (8/103).

The carriage rates of ARGs and point mutations varied across the nine cities in Guizhou (Figure 3B). Isolates from Qianxinan displayed a distinct profile, with the highest rates of *bla_*CTX*–*M*–64_* (4/7, 57.1%), *mphA*/*mrx* (4/7, 57.1%), *oqxA* (3/7, 42.9%), and *qnrS2* (4/7, 57.1%), consistent with their elevated resistance to 3GCs, azithromycin, and fluoroquinolones. The *qnrS1* gene was detected in all nine cities, whereas *qnrS2* was restricted to Qianxinan (4/7, 57.1%), Bijie (2/8, 25.0%), and Qiandongnan (1/7, 14.3%). Isolates from Guiyang carried a unique ESBL gene, *bla_*CTX*–*M*–125_* (2/14, 14.3%), and two *gyrA* mutations (D87Y and D87G) not found elsewhere. Isolates from Tongren exhibited the greatest diversity of ARGs, harboring genes such as *aadA5*, *bla_*TEM*–32_*, and *fosA3* that were absent elsewhere.

**FIGURE 4:**
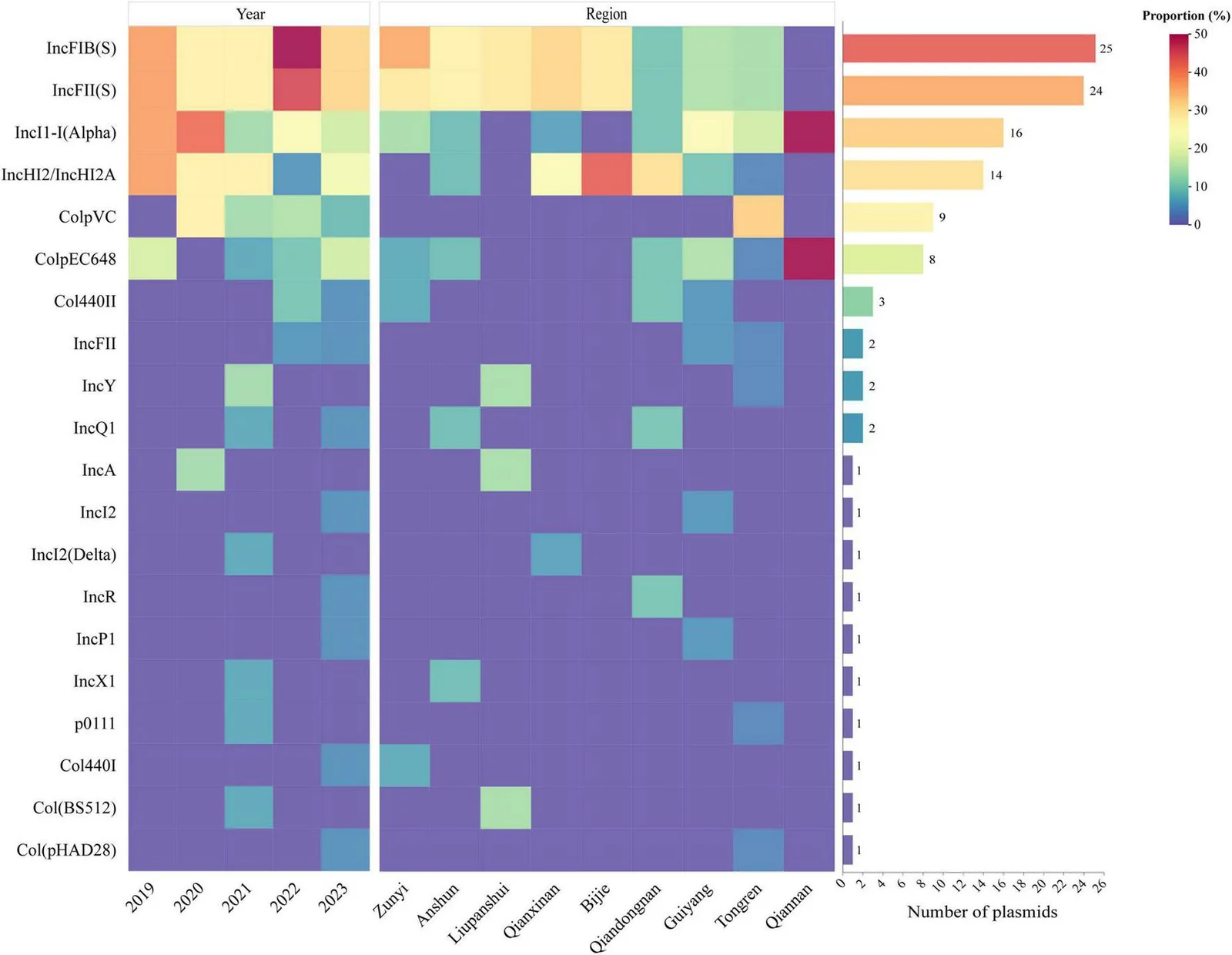
Prevalence of plasmids in *S*. Typhimurium from Guizhou Province. The heatmap depicts the temporal and regional distributions of different plasmid replicons. A bar chart on the right summarizes the counts of each replicon type.

A total of twenty plasmid replicons were identified among 103 *S*. Typhimurium isolates (Figure 4), with an overall carriage rate of 65.0% (67/103) (Supplementary Data S8). IncFIB(S) (25/103, 24.3%) and IncFII(S) (24/103, 23.3%) were the most common, followed by IncI1-I(Alpha) (16/103, 15.5%). The variety of plasmid replicons increased year by year from 2019 to 2023 (from 5 to 14 types). Isolates from Tongren presented the highest diversity (10 types), followed by those from Guiyang (9 types). The dominant plasmid replicons also exhibited regional variation: IncHI2/IncHI2A was most common in Bijie and Qiandongnan; IncI1-I(Alpha) was predominant in Guiyang, Tongren, and Qiannan; IncFIB(S) and IncFII(S) were dominant in the remaining four cities.

### 3.4 Consistency analysis of resistance phenotypes and genotypes

The consistency between resistance phenotypes and genotypes was assessed for seven antibiotics across all 103 isolates (Table 2). Overall, *Kappa* values ranged from 0.16 to 0.74, with six antibiotics demonstrating good agreement and ciprofloxacin showing poor agreement.

**TABLE 2:** Consistency analysis of resistance phenotypes and genotypes of 103 *S*. Typhimurium isolates from Guizhou Province.

Antibiotic class	Antibiotic	Antimicrobial resistance genes (ARGs)	Number of isolates				Sensitivity (%), (95% *CI*)	Specificity (%), (95% *CI*)	*Kappa* (95% *CI*)
			PT:R		PT:S				
			GT: R	GT: S	GT: R	GT: S			
Fluoroquinolones	CIP	*gyrA*(D87N), *gyrA*(D87Y), *gyrA*(D87G), *qnrS1*, *qnrS2*, *qnrB4*, *oqxA*, *oqxB*	17	0	55	31	100.0 (80.5–100.0)	36.0 (26.0–47.1)	0.16 (0.08–0.24)
Macrolides	AZM	*mphA*, *mrx*	8	5	0	90	61.5 (31.6–86.1)	100.0 (96.0–100.0)	0.74 (0.52–0.95)
Third-generation cephalosporins	CAZ	*bla_*CTX*–*M*–14_*, *bla_*CTX*–*M*–55_*, *bla_*CTX*–*M*–64_*, *bla_*CTX*–*M*–65_*, *bla_*CTX*–*M*–125_*, *bla_*CMY*–2_*, *bla_*DHA*–1_*, *bla_*OXA*–10_*, *bla_*TEM*–32_*	13	7	10	73	65.0 (40.8–84.6)	88.0 (79.0–94.1)	0.50 (0.30–0.71)
Penicillin	AM	*bla_*CTX*–*M*–14_*, *bla_*CTX*–*M*–55_*, *bla_*CTX*–*M*–64_*, *bla_*CTX*–*M*–65_*, *bla_*CTX*–*M*–125_*, *bla_*CMY*–2_*, *bla_*DHA*–1_*, *bla_*OXA*–1_*, *bla_*OXA*–10_*, *bla_*TEM*–1_*, *bla_*TEM*–32_*, *LAP-2*	88	6	0	9	93.6 (86.6–97.6)	100.0 (66.4–100.0)	0.72 (0.51–0.93)
Phenicols	C	*floR*, *cmlA1*, *cmlA5*, *cmlA6*, *catB3*	74	9	3	17	89.2 (80.4–94.9)	85.0 (62.1–96.8)	0.67 (0.49–0.84)
Sulfonamides	SXT	*sul1*, *sul2*, *sul3*, *dfrA12*, *dfrA14*, dfrA27*	70	13	2	18	84.3 (74.7–91.4)	90.0 (68.3–98.8)	0.62 (0.44–0.79)
Tetracylines	TE	*tet(A)*, *tet(B)*, *tet(M)*, *tetR*	80	9	5	9	89.9 (81.7–95.3)	64.3 (35.1–87.2)	0.48 (0.25–0.72)

R, resistant; S, susceptible; PT, phenotype; GT, genotype; *CI*, confidence interval. An isolate was classified as GT: R if at least one of the listed genes or mutations was detected.

* For sulfonamides, GT: R required the co-occurrence of ≥ 1 *sul* and ≥ 1 *dfrA* gene. CIP, ciprofloxacin; AZM, azithromycin; CAZ, ceftazidime; AM, ampicillin; C, chloramphenicol; SXT, trimethoprim-sulfamethoxazole; TE, tetracycline.

Among first-line antibiotics, azithromycin displayed good agreement (*Kappa* = 0.74; 95% CI, 0.52–0.95), with a sensitivity of 61.5% (31.6%–86.1%) and a specificity of 100.0% (96.0%–100.0%). Ceftazidime also exhibited good agreement (*Kappa* = 0.50; 95% *CI*, 0.30–0.71). In contrast, ciprofloxacin had the lowest *Kappa* value of 0.16 (0.08–0.24), with a sensitivity of 100.0% (80.5–100.0%) but a specificity of only 36.0% (26.0–47.1%). Specifically, 55 of the 86 phenotypically susceptible isolates carried at least one fluoroquinolone resistance gene.

All four conventional antibiotics showed good phenotype–genotype agreement. Ampicillin showed the highest concordance (*Kappa* = 0.72; 95% CI, 0.51–0.93), followed by chloramphenicol (*Kappa* = 0.67; 95% *CI*, 0.49–0.84). The *Kappa* values for sulfonamides and tetracycline were 0.62 (95% *CI*, 0.44–0.79) and 0.48 (95% *CI*, 0.25–0.72), respectively.

No resistance to ertapenem, meropenem, or tigecycline was observed, and no corresponding carbapenemase or tigecycline resistance genes were detected. Notably, one colistin-resistant isolate (SM2021076) was identified but harbored no *mcr* genes.

### 3.5 Prediction of ARGs and point mutations on plasmids and chromosomes

**FIGURE 5:**
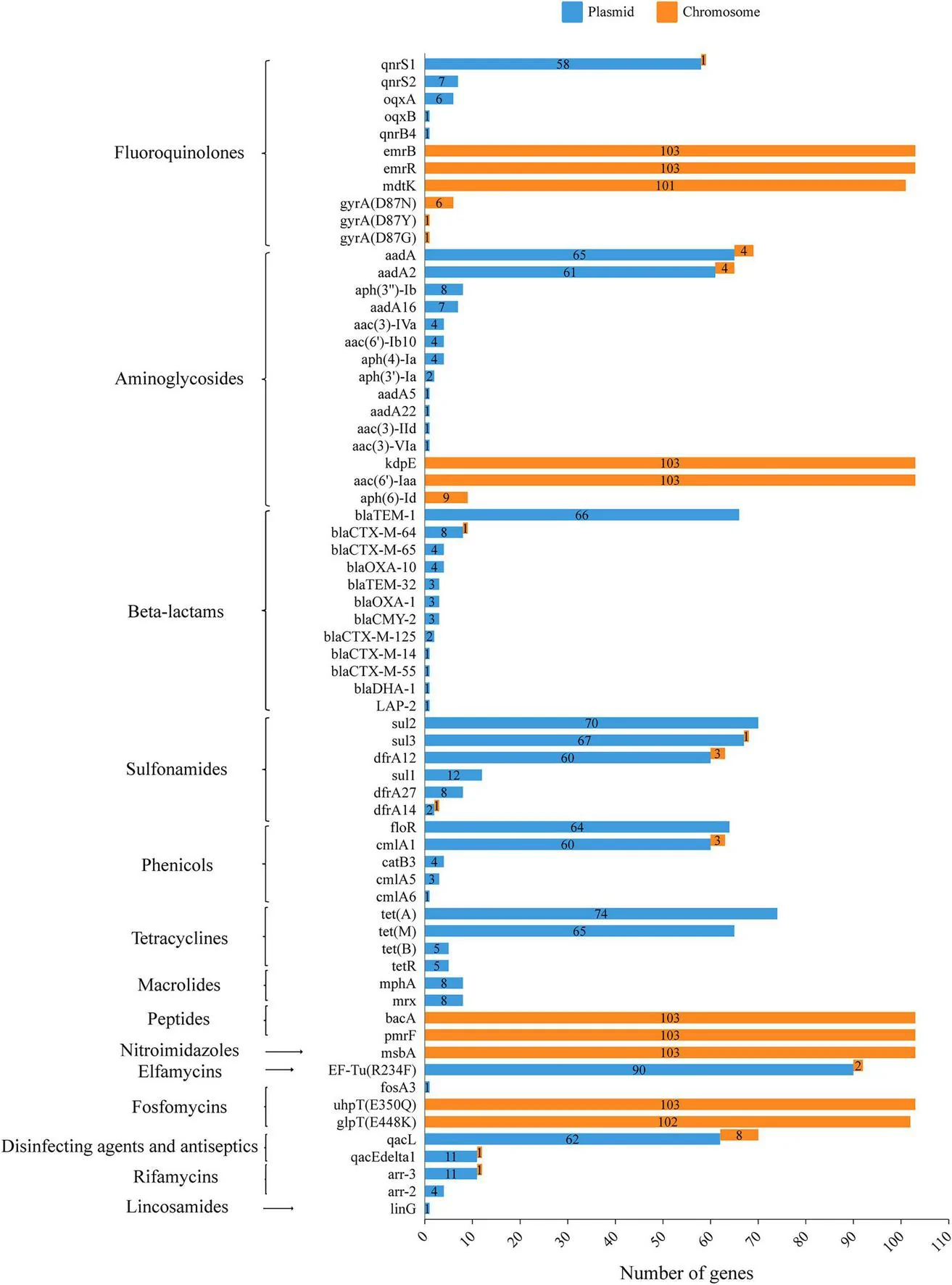
Prediction of ARGs and point mutations on plasmids and chromosomes of 103 *S*. Typhimurium isolates in Guizhou Province.

*In silico* genomic localization analysis revealed that 52 ARGs and a single point mutation were predicted to be located on plasmid-associated contigs in *S*. Typhimurium isolates from Guizhou Province. Among these, the fluoroquinolone resistance gene *qnrS1*, the aminoglycoside resistance gene *aadA*, the penicillin resistance gene *bla_*TEM*–1_*, the sulfonamide resistance gene *sul2*, the chloramphenicol resistance gene *floR*, and the tetracycline resistance gene *tet(A)* were the most common within their respective classes (Figure 5). Furthermore, most ARGs conferring resistance to first-line antibiotics were predicted to be located on plasmid-associated contigs, specifically fluoroquinolones (*qnrS1*, *qnrS2*, *oqxA*, *oqxB*, and *qnrB4*), penicillins (*bla_*TEM*–1_*), 3GCs (*bla_*CTX*–*M*–14_*, *bla_*CTX*–*M*–55_*, *bla_*CTX*–*M*–64_*, *bla_*CTX*–*M*–65_*, *bla_*CTX*–*M*–125_*, and *bla_*CMY*–2_*), and macrolides (*mphA*/*mrx*). For the point mutations, only the elfamycin mutation *EF-Tu* (R234F) (90/103, 87.4%) was predicted to be plasmid-associated.

Conversely, 20 ARGs and six point mutations were predicted to be located on chromosome contigs. Among these ARGs, those conferring resistance to fluoroquinolones (*emrB* and *emrR*), aminoglycosides [*kdpE* and *aac(6’)-Iaa*], peptides (*bacA* and *pmrF*), and nitroimidazoles (*msbA*) were all predicted to be chromosome-associated. For point mutations, three fluoroquinolone resistance mutations in *gyrA* (D87N, D87Y, and D87G) and two fosfomycin resistance mutations in *glpT* (E448K) and *uhpT* (E350Q) were all predicted to be located on chromosome contigs.

### 3.6 CgMLST analysis of *S.* Typhimurium

#### 3.6.1 The prevalence of cgMLST

**FIGURE 6:**
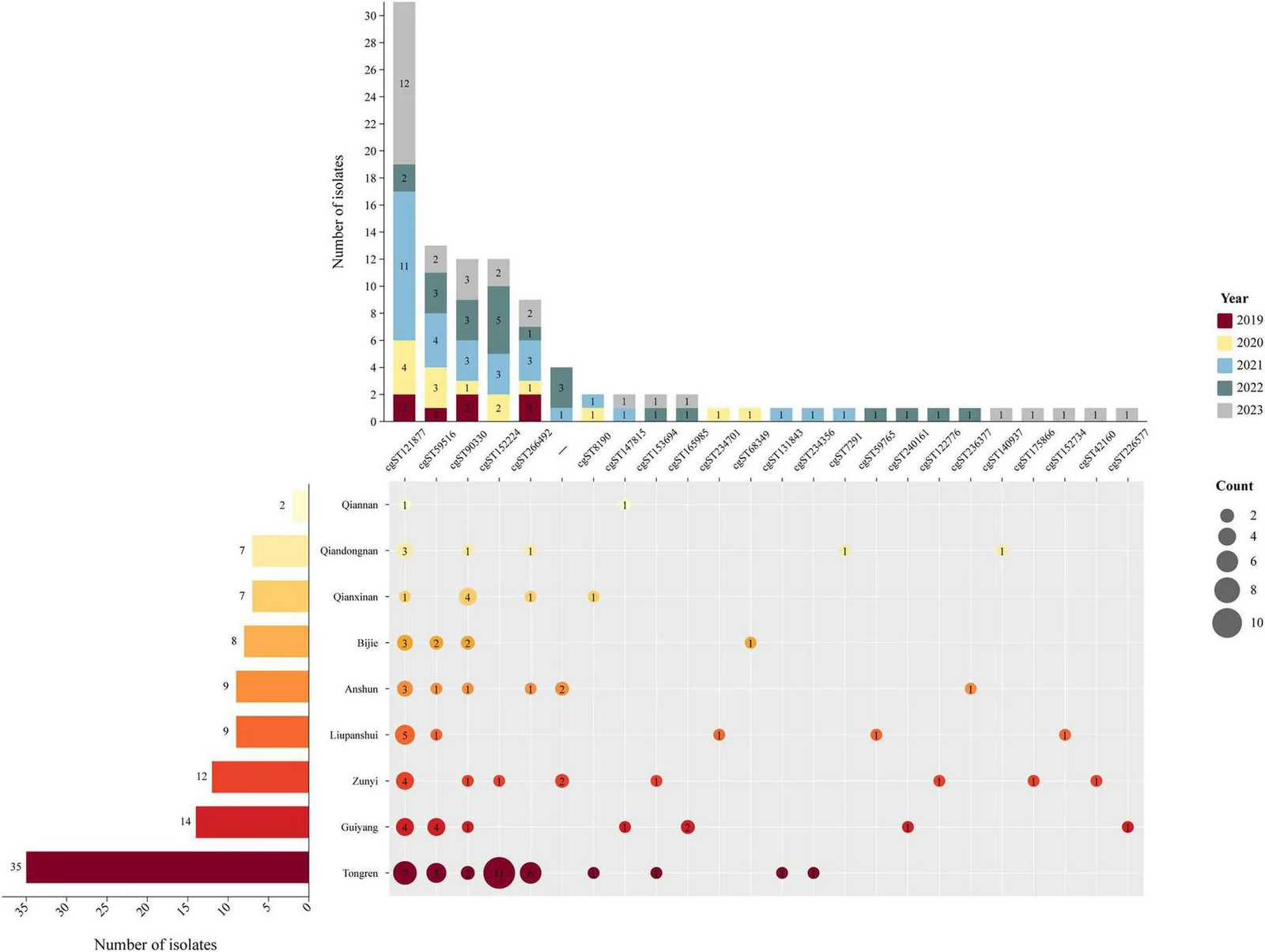
Molecular epidemiological analysis of *S*. Typhimurium in Guizhou Province. The stacked bar chart at the top shows the temporal distribution of cgSTs. The bubble plot below details the distribution of cgSTs among isolates from nine cities (prefectures). The vertical bar chart on the left summarizes the number of isolates.

The cgMLST analysis revealed that 103 *S*. Typhimurium isolates from Guizhou were divided into 23 cgSTs (Figure 6). Among these, cgST121877 (31/103, 30.1%) was the most common, followed by cgST59516 (13/103, 12.6%), cgST90330 (12/103, 11.7%), and cgST152224 (12/103, 11.7%). The diversity of cgST among *S*. Typhimurium isolates increased annually, rising from four in 2019 to 13 in 2023. CgST121877 was the most common cgST in all years except 2022. In that year, cgST152224 became the most prevalent (5/23, 21.7%). A similar trend was observed for the second common cgST: cgST59516 held this position in all years except 2023, when it was replaced by cgST90330 (3/29, 10.3%). Additionally, isolates from Tongren exhibited the highest diversity (9 cgSTs), followed by Zunyi (8 cgSTs) and Guiyang (7 cgSTs). While cgST152224 (11/35, 31.4%) and cgST90330 (4/7, 57.1%) were the most common cgSTs in Tongren and Qianxinan, respectively, cgST121877 remained dominant in the remaining seven cities (prefectures).

#### 3.6.2 Phylogenetic analysis of *S*. Typhimurium based on cgMLST

**FIGURE 7:**
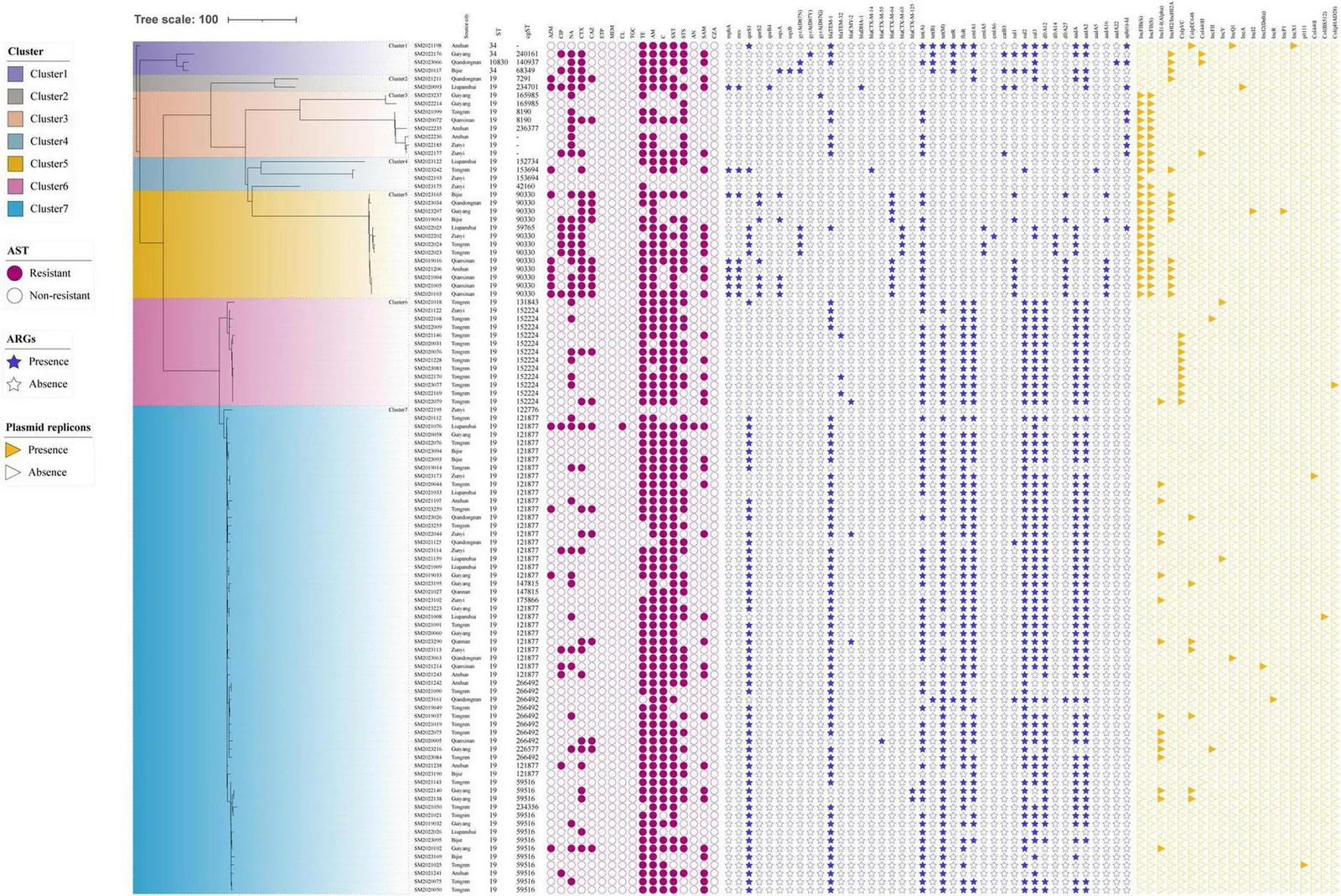
Phylogenetic analysis of 103 *S*. Typhimurium isolates based on cgMLST. The NJ tree was constructed using cgMLST alleles, with branch colors representing the seven distinct clusters (1-7). Three heatmaps on the right detail the distributions of resistance phenotypes, ARGs, and plasmid replicons. Antibiotic abbreviations in the resistance phenotype heatmap are labeled as follows: AZM, azithromycin; CIP, ciprofloxacin; NA, nalidixic acid; CTX, cefotaxime; CAZ, ceftazidime; ETP, ertapenem; MEM, meropenem; CL, colistin; TGC, tigecycline; TE, tetracycline; AM, ampicillin; C, chloramphenicol; SXT, trimethoprim-sulfamethoxazole; STS, streptomycin; AN, amikacin; SAM, ampicillin/sulbactam; CZA, ceftazidime/avibactam.

Phylogenetic analysis based on cgMLST categorized 103 *S*. Typhimurium isolates into seven distinct clusters (Figure 7). Most (99/103, 96.1%) of isolates clustered within Clusters 2–7 and belonged to ST19, suggesting that ST19 is a dominant clonal lineage in this region. In contrast, only four isolates clustered in Cluster 1, mostly belonging to ST34. This cluster was genetically divergent from the ST19 lineage, displayed MDR phenotypes, and carried various ARGs [such as *tet(B)* and *sul3*].

Isolates within Clusters 3–5 were recovered across all 5 years and distributed in eight cities (prefectures) of Guizhou. Notably, the macrolide resistance genes *mphA* and *mrx* mainly clustered within Clusters 3–5 (7/8, 87.5%), and isolates carrying these genes exhibited resistance to azithromycin. IncFIB(S) and IncFII(S), the two most prevalent plasmid replicons among *S*. Typhimurium isolates in Guizhou, were all clustered within Clusters 3–5. In Cluster 5, the isolates were collected across all 5 years and widely distributed across eight cities (prefectures) of Guizhou. Additionally, the most common cgST of isolates in Cluster 5 was cgST90330 (12/13, 92.3%). Analysis integrating resistance genotypes and phenotypes further revealed that isolates in Cluster 5 all carried the *bla_*CTX*–*M*_* gene and were resistant to cefotaxime. Compared with other Clusters, isolates in Cluster 5 exhibited a greater diversity of fluoroquinolone resistance genes (*qnrS1*, *qnrS2*, *oqxA*) and a higher prevalence of macrolide resistance genes (*mphA* and *mrx*). In addition, the carriage rate of IncHI2/IncHI2A plasmids in this cluster was higher (9/13, 69.2%).

Cluster 6 was mainly composed of cgST152224 isolates, with the vast majority (11/12, 91.7%) originating from Tongren. The isolates within Cluster 6 carried multiple ARGs conferring resistance to conventional antibiotics, including *tet(M)* (tetracycline), *floR* and *cmlA1* (chloramphenicol), *sul2*, *sul3*, and *dfrA12* (sulfonamides), as well as *aadA* and *aadA2* (aminoglycosides). Accordingly, they exhibited high resistance to tetracycline, chloramphenicol, trimethoprim-sulfamethoxazole, and streptomycin. The most common plasmid replicon carried by these isolates was ColpVC.

The majority of isolates (59/103, 57.3%) clustered within Cluster 7, with cgST121877 and cgST59516 being the most prevalent. These two subtypes were recovered across all 5 years and distributed in nine cities (prefectures) of Guizhou. Despite originating from different regions, the isolates clustered tightly on the cgMLST tree, exhibiting close genetic relatedness.

Additionally, Cluster 7 was significantly associated with a higher carriage of the fluoroquinolone resistance gene *qnrS1* and the penicillin resistance gene *bla_*TEM*–1_*. One isolate (SM2021076) exhibited phenotypic resistance to colistin despite the absence of colistin resistance genes (such as *mcr* variants). Plasmid analysis showed that isolates within Cluster 7 carried the widest variety of plasmid replicons (10 types), with IncI1-I(Alpha) having the highest carriage rate (25.4%).

#### 3.6.3 MST analysis based on cgMLST

**FIGURE 8:**
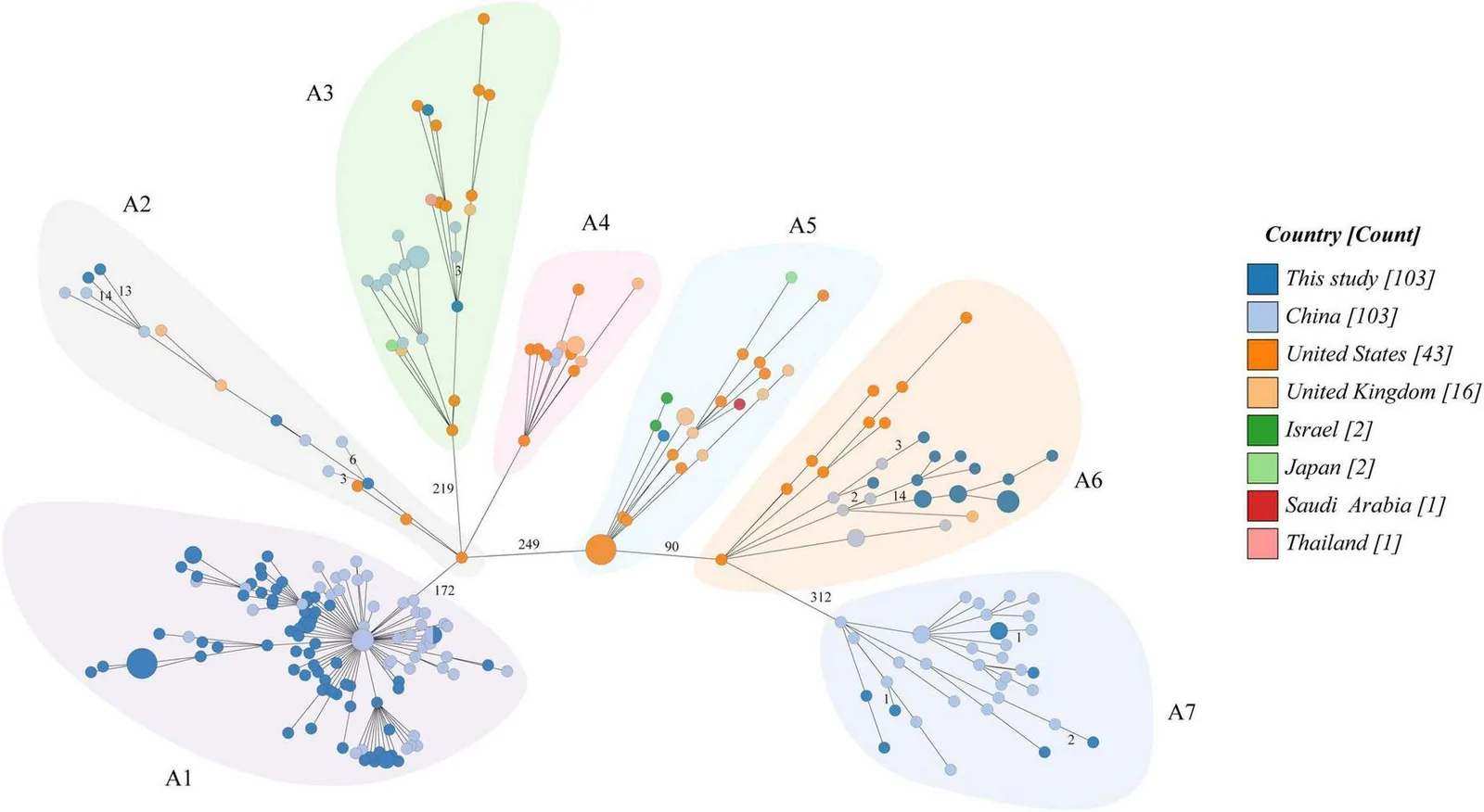
MST based on cgMLST analysis of 271 *S*. Typhimurium genomes. The tree was constructed using the MSTreeV2 algorithm in GrapeTree based on 3,002 core genome allele profiles, including 103 genomes from Guizhou and 168 genomes downloaded from NCBI. A total of 271 *S*. Typhimurium isolates were grouped into seven clusters (A1-A7). The length of the connecting lines represents the number of target genes with different alleles.

To explore the genetic relationships of *S*. Typhimurium in Guizhou Province, an MST was constructed based on cgMLST using 168 publicly available genomes from NCBI and 103 genomes from Guizhou (Figure 8). The 271 isolates were grouped into seven distinct clusters (A1-A7). The majority of isolates (72/103, 69.9%) from Guizhou grouped within cluster A1, where neighboring isolates differed by fewer than 28 alleles. The two dominant cgSTs in Guizhou Province, cgST121877 and cgST59516, were both clustered within the A1 cluster. Among them, an isolate from Liupanshui (SM2021076) differed by only one allele from a pork-derived isolate from Guangxi Province (SH18SF098), and one isolate from Tongren (SM2020075) differed by one allele from a human-derived isolate from Beijing (2019-0952-F).

Except for cluster A4, isolates from Guizhou were sporadically distributed across the remaining clusters and clustered with isolates from the United States and the United Kingdom. Notably, all cgST90330 isolates carried multiple ARGs conferring resistance to first-line antibiotics, and they clustered within A6. Among these isolates, one isolate from Bijie (SM2019054) differed from a duck-derived isolate from Guangxi Province (SH17SF1613) by 14 alleles. Another isolate from Tongren (SM2023242) differed from a human-derived isolate from Shandong Province (WF310) by 3 alleles. In clusters A2 and A7, many isolates from Guizhou exhibited short genetic distances to isolates from other regions of China. In cluster A2, one isolate from Guiyang (SM2022176) differed by 3 alleles from a human-derived isolate in Beijing (2022-0272-NF). Two isolates from Guizhou, one from Qiandongnan (SM2023066) and one from Bijie (SM2020117), differed by 13 and 14 alleles, respectively, from a pork-derived isolate from Hubei Province (SH18SF1029). In cluster A7, two isolates (SM2021099 from Tongren and SM2020072 from Qianxinan) both belonged to cgST8190 and differed by only one allele from a duck-derived isolate from Yunnan Province (SH18SF1601). One isolate from Anshun (SM2022236) differed from a human-derived isolate from Zhejiang Province (SA838) by one allele.

### 3.7 CgSNP analysis

**FIGURE 9:**
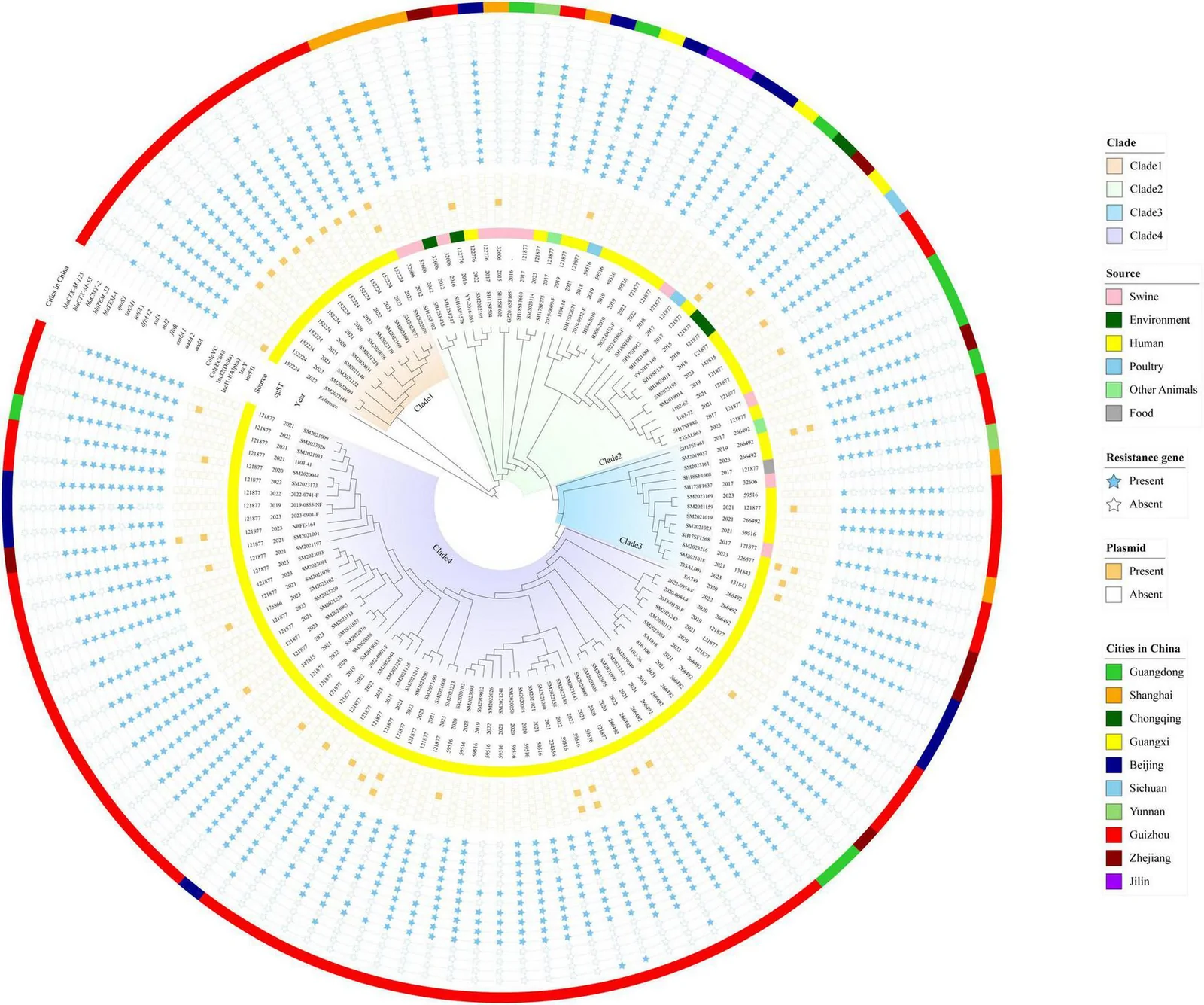
The ML phylogenetic tree of 118 *S.* Typhimurium genome sequences (including 72 from Guizhou Province and 46 downloaded from NCBI) was constructed using cgSNP. *S*. Typhimurium LT2 served as the reference genome, and branch colors represent different Clades. Epidemiological information for each isolate is displayed from the inner circle to the outer circle, and the features represented are as follows: 1. Year of isolation, 2. cgST, 3. Source of isolation, 4. Plasmid replicons, 5. ARGs, 6. Cities in China.

To further investigate the evolutionary relationships of *S*. Typhimurium from Guizhou Province, we performed a cgSNP analysis on cluster A1 (including 72 isolates from Guizhou and 46 isolates from other regions of China) in the MST (Figure 9). The 118 isolates were categorized into four distinct clades: Clade 1 (12 isolates, 10.2%), Clade 2 (32 isolates, 27.1%), Clade 3 (13 isolates, 11.0%), and Clade 4 (61 isolates, 51.7%). Isolates from Guizhou were distributed across all four clades, with the majority clustering in Clade 1 (12/72, 16.7%) and Clade 4 (48/72, 66.7%).

Isolates in Clade 1 were collected between 2020 and 2023. Except for one isolate from Zunyi, the others (11/12, 91.7%) originated from Tongren and belonged to cgST152224. In this clade, two isolates from Tongren (SM2022169 and SM2022170) differed by 11 SNPs. Additionally, the isolate from Zunyi (SM2021122) differed by 51 SNPs from a Tongren isolate (SM2022009).

Isolates in Clade 4 were recovered across all 5 years and distributed across nine cities (prefectures) in Guizhou. The most common cgSTs in Clade 4 were cgST121877 (35/61, 57.4%), which was also the most prevalent among *S*. Typhimurium isolates in Guizhou. Analysis of ARGs and plasmid replicons revealed that isolates in Clade 4 showed a higher prevalence of the fluoroquinolone resistance gene *qnrS1* and carried a greater variety of plasmid replicon types. Further analysis of genetic relationships within Clade 4 showed that one isolate from Tongren (SM2020044) differed by 18 SNPs from an isolate from Zunyi (SM2023173), and two isolates from Guiyang (SM2022138 and SM2022140) differed by 10 SNPs. In addition, many isolates from Guizhou exhibited close genetic distances to human-derived isolates from other regions of China. For instance, one isolate from Tongren (SM2019049) differed by 48 SNPs from a human-derived isolate from Beijing (2019-0379-F). Another isolate from Zunyi (SM2023173) differed by 48 SNPs from a human-derived isolate from Zhejiang Province (NBFE-164); both were collected in 2023 and belonged to cgST121877.

In Clades 2-3, isolates from Guizhou clustered with isolates from other regions of China, and the sources of these isolates were diverse. In Clade 2, a human-derived isolate from Tongren (SM2019014) differed by 49 SNPs from a pork-derived isolate from Yunnan Province (SH18SF1610), and they had a similar AMR pattern. One isolate from Zunyi (SM2022195) differed by 56 SNPs from a pork-derived isolate from Beijing (SH17SF504), and they both belonged to cgST122776. In Clade 3, one human-derived isolate from Liupanshui (SM2021159) differed by 69 SNPs from a food-derived isolate from Yunnan Province (SH18SF1608). They both belonged to cgST121877 and had a similar AMR pattern.

## 4 Discussion

*Salmonella* remains a major foodborne pathogen characterized by rapidly escalating antimicrobial resistance, leading to its inclusion in the WHO 2024 *Bacterial Priority Pathogens List* (Carroll et al., 2021; Sati et al., 2025). Among its serovars, *S*. Typhimurium has demonstrated an increasing trend in both infection incidence and MDR in recent years (Chen et al., 2023), imposing a substantial burden on clinical diagnosis and therapeutic efficacy. In this study, we retrospectively analyzed the distribution of *S*. Typhimurium isolates in Guizhou Province from 2019 to 2023. The largest numbers of *S*. Typhimurium isolates were observed in Tongren, Guiyang, and Zunyi, which may be partly attributable to higher overall *Salmonella* isolation volumes in these regions. Notably, the proportion of *S*. Typhimurium among all *Salmonella* isolates was highest in Bijie (24.2%), Qianxinan (21.9%), and Anshun (20.0%), indicating that the relative burden of this serovar varies across the province. These differences highlight the value of serovar-specific surveillance for understanding local *Salmonella* epidemiology.

Phenotypic analysis identified persistently high resistance to ampicillin, tetracycline, chloramphenicol, and trimethoprim-sulfamethoxazole over the past 5 years. The sustained dominance of MDR isolates, together with the emergence of XDR isolates, has severely compromised therapeutic efficacy and may contribute to prolonged disease courses and increased mortality. Resistance rates to ciprofloxacin, ceftazidime, and cefotaxime were 16.5, 19.4, and 36.9%, respectively. Among them, the resistance rate to ciprofloxacin was lower than that reported in Henan Province (Wu et al., 2024) but higher than that in Xizang (Wang Y. et al., 2025). Resistance to ceftazidime and cefotaxime exceeded levels reported in Jiangxi Province (Yan et al., 2025) and Western countries (Gao et al., 2023). Additionally, the resistance to azithromycin in Guizhou was 12.6%, which is higher than the 2.6% reported in Hangzhou (Zheng et al., 2024) but considerably lower than the 100% (23/23 isolates) reported among *S*. Typhimurium isolates from human infections in Italy (Peruzy et al., 2025), highlighting regional differences in macrolide resistance. One isolate (SM2021076) exhibited phenotypic resistance to colistin, with a confirmed MIC of 4 μg/mL upon repeat testing, but no plasmid-mediated *mcr* genes were detected. In *Salmonella*, chromosomal mutations in the *mgrB*, *phoPQ*, and *pmrAB* regulatory systems are known to confer colistin resistance by modifying the lipid A moiety of lipopolysaccharide (LPS) (Ricci et al., 2020; Lippa and Goulian, 2009), thereby reducing the binding affinity of colistin. Therefore, the observed resistance in this isolate is likely attributable to chromosomal mutations rather than plasmid-encoded *mcr* genes. Further studies are needed to elucidate the precise mechanism. At the regional level, resistance profiles varied across the nine cities (prefectures) in Guizhou. Isolates from Qianxinan stood out with markedly higher resistance to 3GCs and azithromycin compared with other regions, a pattern reflected in its dominant AMR patterns, which uniquely included cefotaxime and ceftazidime. In contrast, isolates from Zunyi showed relatively lower resistance to conventional antibiotics, and their MDR rate (75.0%) was the lowest among all nine cities. These intra-provincial disparities suggest that local antibiotic use practices and ecological factors may shape region-specific resistance profiles, even within a single province. Intriguingly, the MDR rate in Guizhou (94.2%) is higher than that reported nationally in a recent study of 1,874 *S*. Typhimurium isolates across China (87.1%), where ST34 predominated and ST19 was less frequent (Sun H. et al., 2025). This suggests regional variation in dominant clones and resistance levels, likely influenced by local antimicrobial practices and ecological reservoirs. Therefore, enhancing international and inter-regional collaboration to facilitate the exchange of antibiotic stewardship expertise will be pivotal in optimizing therapeutic efficacy globally and safeguarding public health. An interesting observation of our study was the decline in resistance rates to multiple antibiotics in 2022, with resistance to azithromycin dropping to zero. This decline coincided temporally with the implementation of COVID-19 control measures in Guizhou. It is possible that travel restrictions reduced exposure to resistant bacteria and that enhanced disinfection practices temporarily hindered the horizontal transfer of ARGs. However, this explanation remains speculative, and the potential contribution of COVID-19-related factors requires further investigation integrating antibiotic consumption, population mobility, and environmental monitoring data. Furthermore, given the small and uneven annual sample sizes (ranging from seven isolates in 2019 to 30 in 2021), the observed fluctuation in resistance rates may reflect random variation or shifts in the distribution of dominant clones rather than a true temporal trend. Future studies with larger and more balanced annual sampling are needed to determine whether these fluctuations represent genuine temporal shifts or stochastic events.

Based on WGS data, our analysis revealed that *S*. Typhimurium isolates from Guizhou Province harbor a diverse array of ARGs, encompassing a broad spectrum of antibiotic classes. Specifically, 61 distinct ARGs were detected across the 103 isolates, revealing an intricate resistome architecture driving local antimicrobial resistance. Highly conserved ARGs, such as the chromosomal genes modulating fluoroquinolone efflux (*emrB*, *emrR*) and the aminoglycoside resistance [*kdpE*, *aac(6’)-Iaa*], were ubiquitous (100% prevalence) in *S*. Typhimurium isolates from Guizhou Province. Their consistent carriage across all isolates indicates that these genes have become integral to the core resistance genetic background of the local *S*. Typhimurium population. This phenomenon may be attributed to long-term antibiotic selection pressure and adaptive evolution within the regional environment (Pereira et al., 2023). Furthermore, several ARGs conferring resistance to commonly used antibiotics also showed high prevalence rates, including the tetracycline resistance gene *tet(A)* (71.8%), sulfonamide resistance genes *sul2* (68.0%) and *sul3* (66.0%), and fluoroquinolone resistance gene *qnrS1* (57.3%). The widespread distribution of these ARGs reflects the sustained selective pressure from the extensive use of tetracyclines and sulfonamides in both clinical and veterinary settings, particularly in regional animal husbandry (Xie et al., 2019).

Further analysis of critically important antibiotics revealed key resistance mechanisms, including enzyme-mediated resistance, target mutations, and efflux pump systems. Regarding β-lactam resistance mechanisms, five distinct ESBL genes *bla_*CTX*–*M*_* were identified with an overall carriage rate of 16.5%. Furthermore, the AmpC gene *bla_*CMY*–2_* was detected (2.9%). These findings indicate that a subset of isolates has developed the capacity to hydrolyze 3GCs, compromising the clinical efficacy of β-lactam antibiotics. In addition, macrolide resistance genes *mphA* and *mrx* were detected at a prevalence of 7.8%. This finding confirms the emergence of macrolide (azithromycin) resistance in the region and underscores a potential risk of treatment failure for intestinal infections, necessitating increased clinical vigilance. Point mutations directly alter the structure of drug targets, resulting in high levels of resistance. For instance, *QRDR* mutations in *gyrA*: D87N (5.8%), D87Y (1.0%), D87G (1.0%). Although the *qnr* gene was highly prevalent among the *S*. Typhimurium isolates, the relatively low frequency of target mutations indicates that fluoroquinolone resistance in Guizhou Province is currently mediated primarily by efflux pumps and modifying enzymes, rather than target mutations. Notably, our WGS analysis also identified high-frequency mutations associated with fosfomycin [*uhpT (E350Q)*, *glpT (E448K)*] and elfamycin [*EF-Tu* (*R234F*)], which were classified as potential resistance determinants by the CARD. However, due to the absence of these two resistant phenotypes in our AST panel, we cannot determine the clinical relevance of these mutations or confirm whether they confer phenotypic resistance in these isolates. This finding highlighted the importance of AST, particularly when WGS may not provide a definitive assessment (Liu et al., 2024). The distribution of ARGs and point mutations also exhibited regional heterogeneity. Isolates from Qianxinan harbored the highest rates of *bla_*CTX*–*M*–64_*, *mphA/mrx*, *oqxA*, and *qnrS2*, consistent with their elevated phenotypic resistance to 3GCs, azithromycin, and fluoroquinolones. In contrast, *qnrS1* was ubiquitous across all nine cities, whereas *qnrS2* was largely confined to three regions (Qianxinan, Bijie, and Qiandongnan), suggesting that the latter may be associated with regionally restricted dissemination events. Isolates from Guiyang uniquely carried *bla_*CTX*–*M*–125_* and two *gyrA* mutations (D87Y and D87G) not found elsewhere, possibly reflecting localized clonal expansion. The regional clustering of specific ARGs highlights the need for geographically targeted surveillance to monitor the emergence and spread of high-risk resistance profiles.

Genotype-phenotype consistency analysis confirmed that WGS reliably predicted phenotypic resistance for most antibiotics tested, with *Kappa* values ranging from 0.48 to 0.74 for six of the seven antibiotics. A notable exception was ciprofloxacin, for which sensitivity reached 100.0% but specificity was only 36.0%. This discrepancy likely reflects that the majority of phenotypically susceptible isolates carried one or more plasmid-mediated quinolone resistance (PMQR) genes, particularly *qnrS1*, without accompanying target mutations in *gyrA* or *parC*. PMQR genes alone frequently confer only low-level resistance that remains below the clinical breakpoint, although they may facilitate the emergence of higher-level resistance under antibiotic pressure (Miranda et al., 2022; Rodríguez Martínez et al., 2016). For azithromycin and ceftazidime, the good agreement between genotype and phenotype supports the utility of WGS as a surveillance tool for detecting resistance to these first-line antibiotics. Collectively, these findings highlight both the strengths and limitations of WGS-based resistance prediction and reinforce the complementary value of phenotypic AST, particularly for fluoroquinolones.

As the primary category of MGEs, plasmids serve as pivotal drivers of genomic evolution in *Salmonella* and are instrumental in disseminating AMR. Plasmid analysis in this study revealed that more than half (65.0%) of the isolates carried at least one plasmid replicon. The most prevalent replicon types were IncFIB(S) (24.3%) and IncFII(S) (23.3%), which were consistent with findings from previous studies (Chen et al., 2022; Lim et al., 2024). IncF-type plasmids, which often carry multiple ARGs and readily disseminate among Enterobacteriaceae, serve as important vectors driving the spread of MDR (Ammar et al., 2024; Pitout and Chen, 2023). Meanwhile, there was an increase in plasmid replicon types (5–14) between 2019 and 2023, a trend that may signal continuous diversification of resistant plasmids and contribute to the expansion of the MGEs reservoir linked to antimicrobial resistance. Additionally, substantial regional disparities in plasmid profiles were evident, which may be associated with multiple regional factors, including local farming practices, patterns of antibiotic use, and the mobility of humans and animals. Our study further revealed that a high diversity and abundance of ARGs were predicted to be located on plasmid-associated contigs, particularly those associated with resistance to commonly used antibiotics. Among these, *qnrS1*, *aadA*, *bla_*TEM*–1_*, *sul2*, *floR*, and *tet(A)* were the most common. Notably, the majority of ARGs conferring resistance to first-line antibiotics were predicted to be plasmid-associated, suggesting a potential for horizontal transfer. The presence of these ARGs associated with plasmids, including various *qnr* genes (fluoroquinolones), *bla_*CTX*–*M*_* and *bla_*CMY*–2_* (3GCs), and *mphA*/*mrx* (macrolides), was consistent with previous reports (She et al., 2023; Tran et al., 2021; Xie et al., 2022). Given that plasmids may serve as key vectors for horizontal gene transfer, the above findings suggest a potential for this resistance to disseminate among bacterial populations through mechanisms such as conjugation or transformation, potentially posing a significant public health threat.

Molecular typing revealed ST19 and its subtypes (cgST121877 and cgST59516) as the most prevalent sequence types of *S*. Typhimurium in Guizhou Province. The close genetic relatedness and wide geographic distribution of these two dominant subtypes in the province suggest marked clonal expansion of this ST19 lineage in the region during the study period. Furthermore, 103 *S*. Typhimurium isolates were divided into 23 cgSTs. The diversity of cgST increased annually from 4 in 2019 to 13 in 2023, which may be associated with multiple microevolutionary events over the five years. Additionally, isolates from more developed cities, such as Tongren, Zunyi, and Guiyang, displayed notable genetic diversity. This pattern may be associated with factors such as greater commercial trade, frequent human mobility, and more complex food supply chains. The escalating genetic diversification of *S*. Typhimurium introduces unprecedented complexities to public health systems, driven by the variable susceptibility landscapes of emerging sequence types to frontline therapeutics. These dynamics underscore the critical necessity for continuous longitudinal surveillance and deep mechanistic insights to design precise, proactive containment protocols.

CgMLST analysis revealed that the distribution of plasmid replicons carried by *S*. Typhimurium isolates exhibited distinct partitioning across phylogenetic clusters. Among them, the IncHI2 plasmid was primarily identified in Cluster 5, which also carried *bla_*CTX*–*M*_* genes, *qnr* genes, and the *mphA* gene. This structural congruence suggests a potential co-evolutionary trajectory linking specific plasmids, resistance determinants, and discrete bacterial clonal lineages. The IncHI2 plasmid is transferable by conjugation among Enterobacteriaceae and often carries insertion sequences (such as IS26) that facilitate the recombination of ARGs (Che et al., 2021; Varani et al., 2021). Additionally, its circulation is associated with reduced susceptibility to last-resort antibiotics such as carbapenems (Song et al., 2023; Wan et al., 2024). The pattern of co-occurrence of *bla_*CTX*–*M*_*, *qnrS*, and *mphA* with IncHI2 plasmids observed in our study is consistent with findings in other *Salmonella* serovars and Enterobacteriaceae globally, raising the possibility that these plasmids play a role in the circulation of MDR genes, pending functional validation of direct transfer events. Similar plasmids carrying ESBL or carbapenemase genes have been reported in foodborne and clinical isolates in other Chinese provinces (Zhan et al., 2025), as well as other countries (Adler et al., 2025), underscoring their significant epidemiological role. Comparisons with international reports indicate that while high MDR prevalence and plasmid-associated ARG circulation are globally observed, specific dominant clones and plasmid profiles vary by region, reinforcing the importance of localized genomic surveillance for effective intervention strategies. IncF-family plasmids [IncFIB(S) and IncFII(S)] were identified in Cluster 3–5. The shared presence of these plasmids with the *mphA/mrx* genes across the same clusters may explain the clonal aggregation of azithromycin-resistant isolates. IncF-family plasmids exhibit high replication efficiency in *S*. Typhimurium and are difficult to eliminate with curing agents, which has been linked to local outbreaks (Oluwadare et al., 2020). The IncI1-I(Alpha) plasmid was primarily in Cluster 7, where *bla_*TEM*–1_* and *qnrS1* genes were also prevalent. Although its association with specific ARGs appears less pronounced compared to the IncHI2 and IncF plasmids, its high carriage rate may suggest a role in bacterial adaptation and circulation. The ColpVC plasmid was primarily in Cluster 6, which also carried ARGs conferring resistance to conventional antibiotics, including *tet(M)*, *floR*, *sul2/3*, and *aadA*. Its increased prevalence among isolates from Tongren may suggest persistent selective pressure from conventional antibiotics in the local environment. The incompatibility group of plasmids can influence the circulation efficiency of ARG and bacterial host adaptability (Jiang et al., 2025; Sun Z. et al., 2025). Notably, the putative roles of these plasmids in ARG mobilization and bacterial adaptation described here are inferred from genomic co-occurrence patterns. While these associations are plausible, they remain genomic inferences rather than directly demonstrated plasmid architectures or confirmed transfer events. The reliance on short-read sequencing in this study limits our ability to definitively resolve full plasmid structures or the precise localization of ARGs. Therefore, future research incorporating long-read sequencing to resolve complete plasmid scaffolds and conjugation assays to experimentally validate transfer potential is essential. Such integrated analysis of plasmid dynamics will be pivotal in developing targeted intervention strategies to curb the circulation of ARGs.

MST analysis categorized the 271 isolates (103 from Guizhou and 168 downloaded from the NCBI) into seven clusters (A1-A7). Isolates from Guizhou were sporadically distributed in the clusters (A2, A3, A5, A6, A7), where they formed genetic clusters with isolates from both international (the United States, the United Kingdom) and domestic (such as Guangxi, Yunnan, Jiangsu, Hubei, Beijing, Shanghai). This shared genomic clustering is consistent with potential interprovincial and transnational genetic relatedness of *S*. Typhimurium lineages across regions. Specifically, in cluster A2, an isolate from Bijie (SM2020117) differed by 14 alleles from a pork-derived isolate (SH18SF1029) from Hubei Province. In cluster A6, one isolate from Bijie (SM2019054) differed from a duck-derived isolate from Guangxi Province (SH17SF1613) by 14 alleles. In cluster A7, an isolate from Tongren (SM2021099) differed by one allele from a duck-derived isolate (SH18SF1601) from Yunnan. These observations raise the possibility of interprovincial or transnational circulation, which may suggest the existence of animal-to-human transmission chains potentially driven by poultry trade or migration (Kang et al., 2022; Wang Z. et al., 2025). However, it is important to note that the present study only included human-derived isolates from Guizhou Province. The isolates from food and animals showing genetic similarity to our clinical isolates were obtained from public databases and originated from other regions. Therefore, while these genetic links indicate the phylogenetic relatedness between specific isolates, they do not confirm specific transmission routes or directionality. In future work, we plan to incorporate locally sourced food and animal isolates from Guizhou to further test this hypothesis and better understand the potential role of the food supply chain in the spread of resistant *S*. Typhimurium. Additionally, previous studies using cgMLST for *Salmonella* outbreak investigations have commonly employed a threshold of ≤ 7 alleles to define clusters of closely related *Salmonella* isolates (Dangel et al., 2019). However, this threshold is not absolute and may vary depending on the genetic diversity of the serovar, the epidemiological context, and the resolution of the cgMLST scheme used. In our MST analysis, the majority (69.9%) of isolates from Guizhou grouped within cluster A1 with neighboring allele differences below 28. Within this cluster, we observed small allele differences between some isolates from Guizhou and other Chinese regions. For example, an isolate from Liupanshui (SM2021076) differed by one allele from a pork-derived isolate from Guangxi Province (SH18SF098), and another isolate from Tongren (SM2020075) differed by one allele from a human-derived isolate from Beijing (2019-0952-F). Critically, genomic clustering based on sequence similarity must be distinguished from inferred epidemiological transmission. These allele differences indicate close genetic relatedness and membership in the same genomic clustering, but they do not confirm direct epidemiological linkage or transmission events. In future studies, we will collect traceback metadata to further clarify potential epidemiological connections. In summary, MST analysis revealed a complex genetic clustering pattern for *S*. Typhimurium in Guizhou, characterized by the coexistence of a predominant local cluster (A1) alongside multiple clusters containing isolates from other regions and countries, providing a foundational reference for monitoring the expansion of specific lineages and offering early warnings for potential outbreaks.

To further investigate the population structure of *S*. Typhimurium isolates in Guizhou Province, cgSNP analysis was conducted for isolates within cluster A1, which categorized 118 isolates (72 from Guizhou and 46 from other Chinese provinces) into four clades. Clade 4 was the largest branch (51.7%), dominated by cgST121877 isolates, distributed across nine cities (prefectures) of Guizhou from 2019 to 2023, suggesting sustained and widespread circulation within the province. Additionally, isolates in Clade 4 exhibited pronounced resistance characteristics, including a high carriage rate of the fluoroquinolone resistance gene *qnrS1* and a high prevalence of the IncI1-I(Alpha) plasmid, which may enhance the spread of AMR. In Clade 4, one isolate from Tongren (SM2020044) differed by 18 SNPs from an isolate from Zunyi (SM2023173), and two isolates from Guiyang (SM2022138 and SM2022140) differed by only 10 SNPs. These genetic linkages among human isolates suggest cross-regional circulation may be associated with human mobility. All isolates in Clade 1 originated from Guizhou Province (2020–2023) and exhibited strong geographical clustering, with 91.7% (11/12) from Tongren. The SNP differences among these temporally clustered isolates, such as 11 SNPs between two isolates (SM2022169/170), suggest ongoing microevolution and potential transmission within the local community. In Clades 2–3, isolates from Guizhou clustered with isolates from other provinces and were derived from diverse sources, including humans, food, and animals. For instance, isolates from Guizhou exhibited short genetic distances to an isolate from food in Yunnan (49 SNPs) and Beijing (56 SNPs). These observations are consistent with potential cross-regional and cross-species circulation involving the food chain and product trade. While SNP thresholds have been widely discussed in the context of outbreak detection, studies emphasize that such thresholds are not absolute and should be interpreted in context with epidemiological data. For instance, Pightling et al. reported that < 21 SNPs support a match between isolates in regulatory contexts (Pightling et al., 2018), while Payne et al. demonstrated that 90% of outbreak-related isolates fell within a 5-SNP cutoff and advocated for dynamic thresholds based on local genetic diversity (Payne et al., 2021). Gymoese et al. further showed that SNP cutoffs vary even within a single serovar, lineage, or clone, with observed distances ranging from 0 to 30 SNPs across different outbreaks, emphasizing the need for case-by-case evaluation (Gymoese et al., 2017). Furthermore, potential confounders (including uneven sampling, incomplete metadata in public databases, widespread clones, and the sharing of plasmids between genetically distinct isolates) may influence the observed clustering patterns. In our study, the SNP distances observed within Guizhou (10–18 SNPs) are consistent with recent regional circulation. The larger distances between Guizhou and other provinces (48–69 SNPs) likely reflect longer temporal and geographical separation, unsampled intermediate strains, and cross-species transmission. These factors do not preclude the existence of epidemiological links, but rather highlight the complexity of cross-regional transmission networks. Collectively, the cgSNP analysis provides valuable insights into the spatiotemporal dynamics of *S*. Typhimurium, reinforcing the urgent need for enhanced surveillance systems and geographically tailored interventions.

In summary, the epidemiology of *S*. Typhimurium in Guizhou Province presents a tripartite public health challenge: the dominance of epidemic clones, an extensive and diverse resistome, and the existence of potentially complex transmission networks. These results necessitate the establishment of an integrated, farm-to-fork surveillance network covering animal farms, slaughterhouses and processing facilities, retail markets, and healthcare institutions. Surveillance should prioritize monitoring the dominant clones (ST19/cgST121877/cgST59516) and high-risk clusters (Clusters 5 and 7). Furthermore, high-resolution cgMLST and SNP analysis techniques should be employed to support rapid source tracking and transmission route investigation of outbreak isolates, enabling targeted interventions in collaboration with agricultural, market regulatory, and public health authorities. While our study presents a comprehensive analysis, it is essential to acknowledge several important limitations. First, our analysis only focused on human-derived isolates collected from sentinel hospitals in Guizhou Province. While these hospitals provide broad geographic coverage, they may not capture the full diversity of *S*. Typhimurium circulating in the community, nor do they represent strains from food, animal, or environmental sources. Therefore, our findings may be subject to sampling bias, and caution is warranted when generalizing the results to the entire province or to non-human reservoirs. Second, a significant technical limitation of this study is the reliance on short-read sequencing. While computational classification tools were employed to predict plasmid-associated contigs, the lack of long-read sequencing prevents the definitive resolution of complete, circularized plasmid structures. Consequently, the localization of ARGs and the mechanistic roles of specific plasmids in MDR dissemination discussed here should be interpreted as plausible genomic inferences based on co-occurrence, requiring future functional validation through conjugation assays or long-read genomic resolution. By addressing these limitations in future research, we can further enhance our understanding of *S*. Typhimurium and develop more effective strategies to prevent and control its spread.

## 5 Conclusion

*S*. Typhimurium in Guizhou Province has evolved into a major MDR pathogen driven by a highly diversified resistome, extensive high plasmid carriage, and distinct spatiotemporal evolutionary dynamics. Its resistance is likely associated with efflux systems, potential horizontal gene transfer involving MGEs, and the incremental acquisition of key resistance determinants. Of particular concern is the widespread accumulation of ARGs associated with resistance to critically important antibiotics, specifically 3GCs and fluoroquinolones, which severely limit treatment options. The high prevalence of high-risk ARGs (such as *bla_*CTX*–*M*_* variants and *qnr* family) poses a severe public health threat due to their acute propensity for horizontal mobilization and clonal circulation.

Furthermore, ST19 and its dominant sublineages (cgST121877 and cgST59516) have firmly established themselves as the predominant epidemic lineages in Guizhou, exhibiting tight genetic relatedness and widespread regional distribution. To neutralize this threat, an integrated “One Health” framework spanning human, animal, and environmental sectors is urgently required. Actionable interventions must include: routine WGS of clinical, food-borne, and livestock-derived isolates for the early interception of high-risk lineages; the enforcement of harmonized, cross-sectoral surveillance programs across the “farm-to-fork” continuum (including livestock farms, slaughterhouses, and retail markets); and the establishment of seamless data-interchange protocols between public health, veterinary, and environmental agencies.

Concurrently, cgMLST and SNP analyses should be employed to enhance surveillance of resistant lineages and support future investigation of potential intersections across the food supply chain, facilitating precision interventions across agriculture, market regulation, and public health agencies. Moreover, fostering the development of interoperable provincial, national, and international genomic databases remains vital. Real-time sharing of resistome and genomic data through these centralized platforms is critical for implementing coordinated cross-border responses to effectively disrupt the dissemination of high-risk clones across regional and national boundaries.

## Data Availability

The genome sequences of the 103 S. Typhimurium isolates from Guizhou Province have been deposited in the National Microbiology Data Center (NMDC) under BioProject NMDC10020480 (https://nmdc.cn/resource/genomics/project/detail/NMDC10020480), with accession numbers NMDC60221822-NMDC60221924. The data are publicly accessible and freely downloadable. Detailed metadata for each isolate, including accession numbers, year, city, and source, are provided in Supplementary Data S9.

## Footnotes

1
https://www.bioinformatics.babraham.ac.uk/projects/fastqc/

2
https://card.mcmaster.ca/analyze/rgi

3
https://github.com/tianrenmaogithub/PlasmidHunter

4
https://cge.food.dtu.dk/services/cgMLSTFinder/

5
http://www.iqtree.org

6
https://github.com/achtman-lab/GrapeTree

7
http://www.baiyi-tech.cn/

8
https://github.com/tseemann/snippy

9
https://github.com/nickjcroucher/gubbins/
